# A multi-tissue multi-omics analysis reveals distinct kineztics in entrainment of diurnal transcriptomes by inverted feeding

**DOI:** 10.1016/j.isci.2021.102335

**Published:** 2021-03-19

**Authors:** Haoran Xin, Fang Deng, Meiyu Zhou, Rongfeng Huang, Xiaogen Ma, He Tian, Yan Tan, Xinghua Chen, Dan Deng, Guanghou Shui, Zhihui Zhang, Min-Dian Li

**Affiliations:** 1 Department of Cardiology and the Center for Circadian Metabolism and Cardiovascular Disease, Southwest Hospital, Third Military Medical University (Army Medical University), Chongqing, 400038, China; 2 Department of Pathophysiology, College of High Altitude Military Medicine, Third Military Medical University (Army Medical University), Chongqing, 400038, China; 3 State Key Laboratory of Molecular Developmental Biology, Institute of Genetics and Developmental Biology, Chinese Academy of Sciences, Beijing, 100101, China

**Keywords:** Animal Physiology, Systems Biology, Metabolomics, Transcriptomics

## Abstract

Time of eating synchronizes circadian rhythms of metabolism and physiology. Inverted feeding can uncouple peripheral circadian clocks from the central clock located in the suprachiasmatic nucleus. However, system-wide changes of circadian metabolism and physiology entrained to inverted feeding in peripheral tissues remain largely unexplored. Here, we performed a 24-h global profiling of transcripts and metabolites in mouse peripheral tissues to study the transition kinetics during inverted feeding, and revealed distinct kinetics in phase entrainment of diurnal transcriptomes by inverted feeding, which graded from fat tissue (near-completely entrained), liver, kidney, to heart. Phase kinetics of tissue clocks tracked with those of transcriptomes and were gated by light-related cues. Integrated analysis of transcripts and metabolites demonstrated that fatty acid oxidation entrained completely to inverted feeding in heart despite the slow kinetics/resistance of the heart clock to entrainment by feeding. This multi-omics resource defines circadian signatures of inverted feeding in peripheral tissues (www.CircaMetDB.org.cn).

## Highlights

• A multi-omics analysis of food entrainment in mouse peripheral tissues
• Inverted feeding rhythm entrains diurnal transcriptomes with distinct kinetics
• Phase kinetics of tissue clocks is conditioned by constant light
• Cardiac metabolism entrains to feeding fast with slow kinetics of the heart clock

## Graphical abstract

**Figure:**
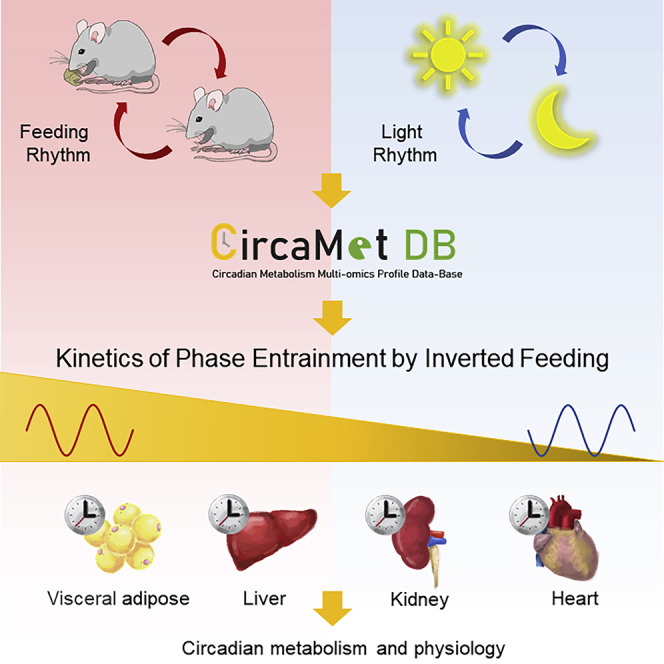

## Introduction

Diurnal oscillations of metabolism, physiology, and behavior is pervasive in life, serving to align the body with environmental cycles and to separate chemically incompatible biochemical reactions (Bass and Lazar, 2016; Takahashi, 2017). Circadian clock enables circadian rhythms of metabolism and physiology in cells, which is reset by daily cycles of two principal environmental cues, such as light and food (Kinouchi and Sassone-Corsi, 2020). Although each cell shares a remarkably similar set of clock genes, tissue clocks are organized in a network and paced by the suprachiasmatic nucleus of the hypothalamus (Dibner et al., 2010; Greco and Sassone–Corsi, 2019). The SCN clock entrains to the light-dark cycle and synchronizes clocks in peripheral tissues, including liver, kidney, heart, and fat tissue, in part through feeding behaviors (Schibler et al., 2016). It is thought that food overrides light in resetting most, if not all, peripheral clocks (Reinke and Asher, 2019). Inverted feeding, also known as daytime-restricted feeding (DRF) or desynchronized feeding, entrains the liver clock to completely inverted oscillations in rodents under light/dark cycles (Damiola et al., 2000; Stokkan et al., 2001).

The coupling between peripheral clocks and feeding rhythms is conducted by nutrient-sensing signaling (Crosby et al., 2019; Hirano et al., 2016; Panda, 2016; Reinke and Asher, 2019; Zhang et al., 2020b). Molecular gears of circadian clocks are primarily made of transcriptional-translational feedback loops, including transcription factors BMAL1, CLOCK, and corepressors PERIOD (PER) and CRY (Patke et al., 2020; Takahashi, 2017). Poly(ADP-ribose) polymerase 1 senses intracellular level of NAD+ and entrains the liver clock to inverted feeding via ploy(ADP-ribosyl)ation of CLOCK (Asher et al., 2010). Glucose-sensing OGT O-GlcNAcylates clock proteins (Yang and Qian, 2017) and modulates diurnal rhythms of voluntary locomotion (Kaasik et al., 2013) and glucose homeostasis (Li et al., 2013). In the past, although many studies focused on clock entrainment in liver, it is not clear whether the regulatory mechanisms of the liver clock are applicable to other peripheral tissues. Emerging evidence suggested that entrainment of peripheral clocks is conditioned by light and non-cell autonomous clocks. The functioning of the liver clock requires the presence of BMAL1-dependent clocks in other tissues under constant darkness (Koronowski et al., 2019), which applies to the epidermal clock (Welz et al., 2019).

Time-restricted feeding (tRF, also known as nighttime-restricted feeding, NRF) is arising as an effective measure to synchronize diurnal oscillations of physiology and metabolism (Longo and Panda, 2016). In liver, circadian clocks control diurnal rhythms of transcriptomes and metabolomes under constant darkness (Krishnaiah et al., 2017; Vollmers et al., 2009). Hepatocyte-autonomous clock contributes to only 20% of diurnal transcriptomes in liver and is sufficient to establish circadian rhythms of glycogen turnover and NAD + salvage metabolism (Koronowski et al., 2019). However, synchronized feed-fast cycles by tRF restores the majority of diurnal transcriptomes in Bmal1^−/−^ mouse liver under light/dark cycles (Greenwell et al., 2019) and protects against obesity and metabolic syndromes in a panel of liver-specific clock mutant mice (Chaix et al., 2019). Provided the salient information on entrainment of liver physiology and metabolism by feeding rhythms, there is a gap in whether and how inverted feeding entrains diurnal oscillations of circadian clocks, physiology, and metabolism in extra-hepatic peripheral tissues.

Here, we applied inverted feeding regimens to uncouple peripheral clocks from the SCN clock and performed 24-h global profiling of transcripts and metabolites in mouse peripheral tissues to study the transition kinetics during inverted feeding, which would transit from the original phase to a 12-h shifted phase if fully entrained by feeding within a week. We first examined entrainment of tissue circadian clocks by feeding and discerned the impact of sex, duration of restricted feeding, and constant light on food entrainment. Next, phase analysis and pathway analysis of diurnal transcriptomes revealed distinct kinetics in food entrainment, which graded from fat tissue, liver, kidney, to heart, and predicted potential physiological and metabolic pathways entrained by inverted feeding. We integrated diurnal transcriptomes with metabolomes/lipidomes and revealed that fatty acid oxidation entrained almost completely to feeding, despite the slow kinetics and/or resistance of the heart clock in food entrainment. In summary, this systems approach sheds light on molecular and metabolic signatures of inverted feeding on circadian biology in peripheral tissues and provides an integrated resource to approach clock synchronization in the body.

## Results

### Global transcript profiling of mouse tissues over 24 h reveals robust diurnal rhythms of transcriptomes under time-restricted feeding

**Figure 1:**
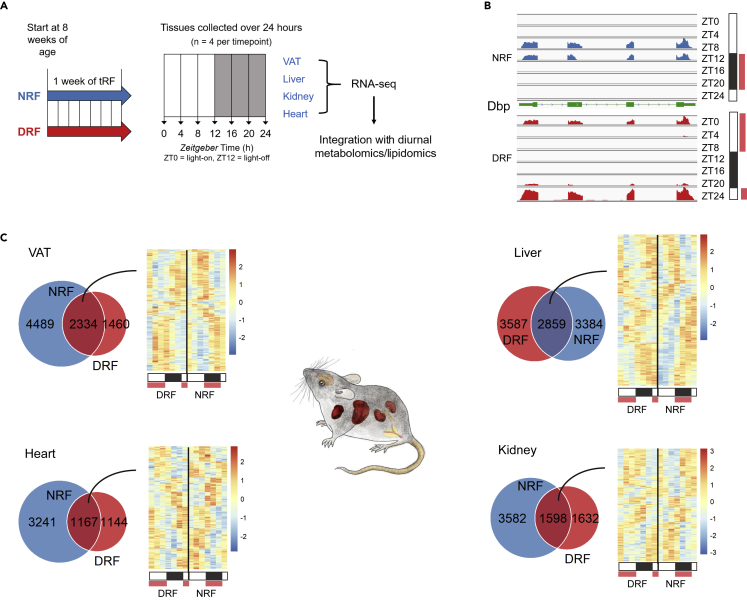
Global transcript profiling of mouse tissues over 24 h reveals robust diurnal rhythms of transcriptomes under time-restricted feeding (A) The workflow of multi-omics analysis of diurnal rhythms in peripheral tissues from female mice subjected to inverted feeding for 1 week. All transcript profiles have at least 40 million reads per sample. DRF, daytime-restricted feeding; NRF, nighttime-restricted feeding; VAT, visceral adipose tissue. See transparent methods. (B) Read counts from the clock output gene *Dbp* locus exhibit diurnal rhythms in liver and are phase inverted under DRF. ZT, zeitgeber time. See also Figures S1A and S1B. (C) General features of the global transcript profiling of peripheral tissues in 9-week-old time-restricted-fed C57BL/6J female mice. Venn diagrams illustrate the overlap of oscillating genes between DRF and NRF in each tissue. Heatmaps show 24-h expression profiles of genes that oscillated under both DRF and NRF conditions.

To study food entrainment of diurnal transcriptomes in peripheral tissues, 9-week-old female mice on time-restricted feeding (tRF) regimens, namely daytime (DRF)- and nighttime (control, NRF)-restricted feeding, for one week were subjected to tissue collection, including visceral adipose tissue (VAT), liver, kidney, and heart, at a 4-h interval over 24 h (Figure 1A). Female mice were chosen because they have not been studied by multi-tissue multi-omics in tRF regimens but are known to respond to inverted feeding (Davidson et al., 2003). This one-week regimen is sufficient to entrain liver clocks in rodents (Damiola et al., 2000; Stokkan et al., 2001). Tissues were subjected to global transcript profiling (Table S1) for an average of 50 million reads with a minimal coverage of 39.6 million reads per sample. Liver, heart, and VAT were further subjected to untargeted metabolomics or targeted lipidomics for integration studies (Figure 1A). In total, we performed 224 deep RNA sequencing (Table S2), 138 untargeted metabolomics, and 48 targeted lipidomics experiments (Tables S3 and S4).

To validate the pipeline, we visualized the Dbp and Arntl (Bmal1) loci from global transcript profiling and confirmed that inverted feeding reversed the phase of Dbp rhythm in liver (Figures 1B and S1A). Inverted feeding for 1 week did not significantly alter the body weight and visceral adiposity in mice (Figure S1B). The effect on body weight is in line with a previous study (Wang et al., 2017). Wheel-running activity recording confirmed that DRF group remained active in the night, which results in a complete phase uncoupling between voluntary locomotor rhythm and feeding rhythm (Figure S1C), which is consistent with a recent study using an automated feeder system (Acosta-Rodríguez et al., 2017). Principal component analysis revealed the effects of tRF regimens on tissue multi-omics (Figure S1D). Expression levels of transcripts were subjected to MetaCycle rhythmicity analysis with Benjamini-Hochberg procedure. About 10%–30% of the transcriptome exhibit diurnal rhythms in peripheral tissues (Table S2). This is consistent with published work (Hughes et al., 2009; Zhang et al., 2014). We detected 1167–2859 diurnal genes that oscillated under both DRF and NRF regimens (Figure 1C, Table S2), which allowed phase analysis to understand transcriptome-wide phase entrainment by inverted feeding in peripheral tissues. In sum, robust diurnal rhythms of tissue transcriptomes under tRF regimens were detected by diurnal transcriptomics.

### Circadian clocks show tissue-specific kinetics in phase entrainment by inverted feeding

**Figure 2:**
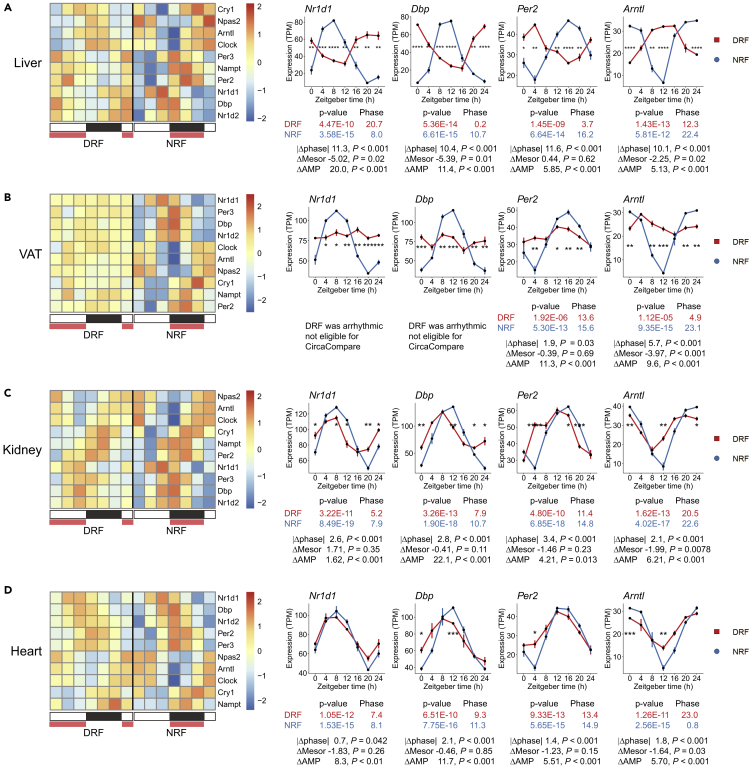
Circadian clocks show tissue-specific kinetics in phase entrainment by inverted feeding (A–D) Diurnal expression of clock genes in liver (A), VAT (B), kidney (C), and heart (D). In heatmaps, expression levels are represented as Z score of the mean (n = 4). White/black blocks indicate light/dark cycle. Red frames indicate feeding period. In line-plots, samples are represented as mean ± sem (n = 4). Statistics and comparison of rhythmicity parameters were computed by CircaCompare. Mesor, rhythm-adjusted mean expression level; AMP, amplitude. Multiple t-tests with Bonferroni correction; not shown when p ≥ 0.05, ∗p < 0.05, ∗∗p < 0.01, ∗∗∗p < 0.001, ∗∗∗∗p < 0.0001. See also Figure S2.

To test whether cell-autonomous circadian clocks entrained to inverted feeding with different kinetics, we examined the expression profiles of clock genes, including Nr1d1 (Rev-Erbα), Dbp, Per2, and Arntl (Bmal1). The statistical significance of changes in rhythmicity parameters was computed by CircaCompare (Parsons et al., 2020). The results showed that inverted feeding completely reversed the phase of clock genes in liver and decreased the amplitude (Figure 2A). This is consistent with previous knowledge (Reinke and Asher, 2019). In VAT, 24-h expression profiles of Nr1d1 and Dbp became arrhythmic under DRF (Figure 2B). Diurnal rhythms of Per2 and Arntl showed incomplete phase shifts by 2 h and 6 h, respectively and exhibited a large decrease of amplitude in VAT (Figure 2B). These observations suggested a dampening response of the adipose clock in phase entrainment by feeding. In kidney (Figure 2C) and heart (Figure 2D), diurnal rhythms of clock genes showed phase shifts of 0–3 h, which suggested slow kinetics and/or resistance in phase entrainment of clocks in these tissues by feeding.

To determine whether the different kinetics in clock-entraining responses to inverted feeding are modulated by sex-related signals, we performed quantitative PCR analysis and quantified diurnal expression profiles of clock genes in male mice that had been subjected to inverted feeding for one week. As expected, diurnal rhythms of clock genes such as Nr1d1, Dbp, and Arntl entrained to inverted feeding almost completely in males (Figure S2A). Diurnal rhythm of Per2 was less robust under DRF, but the peak time as roughly 12 h apart between tRF. In VAT, diurnal rhythms of Nr1d1 and Dbp were dampened by inverted feeding (Figure S2B). Unlike in female VAT, diurnal rhythms of Per2 became dampened, whereas that of adipose Arntl was reversed by inverted feeding in male VAT (Figure S2B). In kidney, diurnal rhythms of Dbp and Arntl were phase-locked under DRF and severely impaired on amplitude (Figure S2C). Diurnal profiles of Nr1d1 and Per2 became arrhythmic under DRF. In heart, diurnal rhythms of clock genes were dampened by inverted feeding (Figure S2D). Together, the liver clock and part of the adipose clock maintained similar responses to inverted feeding between male and female mice; however, peripheral clocks in kidney and heart seemed to be less robust under DRF in males.

**Figure 3:**
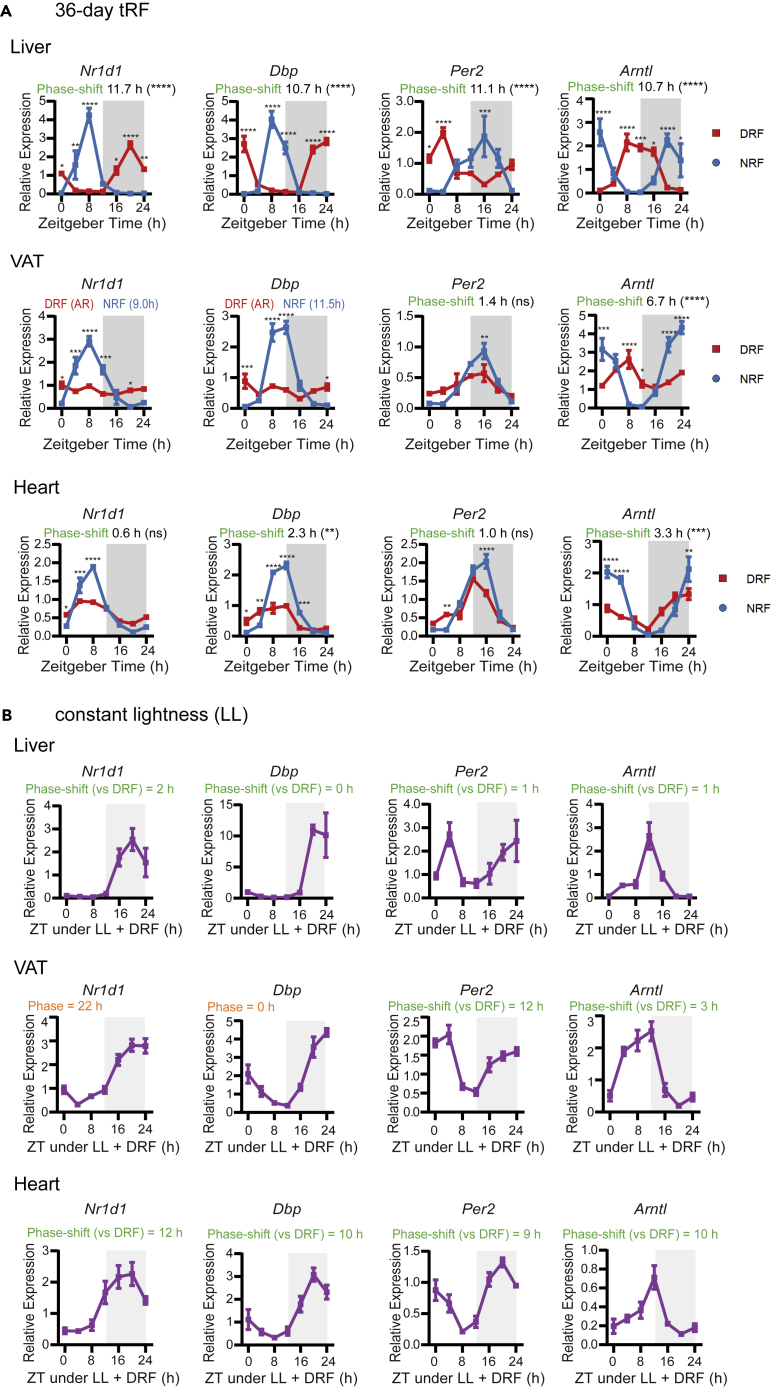
Effects of duration and constant light on clock entrainment by inverted feeding (A) Effects of 36-day inverted feeding on diurnal expression of clock genes in liver, VAT, and heart. Phase shifts were computed by CircaCompare, with statistical significance from t tests indicated in the brackets. Phase parameter from MetaCycle (meta2d_phase) was shown when CircaCompare could not be applied. ns, p ≥ 0.05, ∗p < 0.05, ∗∗p < 0.01, ∗∗∗p < 0.001, ∗∗∗∗p < 0.0001. See also Figures S3A–S3C. (B) Effects of constant light (LL) on diurnal expression of clock genes in liver, VAT, and heart under DRF. Female mice were acclimated to 12-h light-dark cycles, subjected to LL for 9 days, and then subjected to DRF and LL for 7 days. Phase shift was estimated by meta2d_phase (H) with the period length under LL set to 20–28 h and that under LD set to 24 h. CircaCompare could not be applied because the period length under LL differs significantly from that under 12-h light-dark cycles. Data are represented as mean ± sem (n = 4). Multiple t tests with Bonferroni correction; not shown when p ≥ 0.05, ∗p < 0.05, ∗∗p < 0.01, ∗∗∗p < 0.001, ∗∗∗∗p < 0.0001. See also Figures S3D–S3E.

To determine whether clock entrainment in peripheral tissues reaches the steady state, we subjected female mice to inverted feeding for 36 days. We observed that body weight decreased slightly and transiently in the first two weeks, restored, and increased later in DRF-treated mice (Figure S3A), which is associated with a transient drop in food intake (Figure S3B). This effect is well powered by a sample size of 56 (a sample size of 26 is required to ensure a power of 0.8 when alpha = 0.05) and thus could reveal the small change of body weight and food intake. Profiling of clock genes in peripheral tissues around the clock revealed that after 36 days under tRF regimens, the phase shifts of circadian clocks were not different from that in the 7-day tRF in metabolic tissues, such as liver and VAT (Figure 3A). Although heart and kidney clocks remained phase-locked to light-dark cycles under 36-day tRF, the amplitude was clearly decreased by inverted feeding (Figures 3A and S3C). We could not rule out the possibility that heart and kidney clocks would eventually shift provided even longer tRF, but at least these peripheral clocks showed resistance to phase entrainment by feeding after 36 days of tRF. Comparison of phase kinetics between 36-day tRF and 7-day tRF clarified that clock entrainment under 7-day tRF regimens reflects tissue-specific kinetics.

Light and food are two principal time cues in the mammalian circadian clock system. To determine whether removal of time cues from the central clock would facilitate phase entrainment of peripheral clocks by feeding, we used constant light (∼500 lux, LL) to desynchronize the SCN clock neurons (Chen et al., 2008; Ohta et al., 2005) and examined phase adaptation to inverted feeding in female mice under LL. Voluntary running activity analysis showed that LL significantly increased the period of behavioral rhythm by 1.7 h, and DRF did not significantly alter the period in these LL-conditioned female mice (Figure S3D, LD: 23.91 ± 0.012 h, LL: 25.61 ± 0.18 h, LL + DRF: 25.24 ± 0.55 h, mean ± sem, n = 8). Twenty-four-hour profiling of clock genes showed that LL did not alter the phase of clock genes in liver, which had already entrained to feeding (Figure 3B).

LL seemed to facilitate non-hepatic tissue clocks to entrain completely to inverted feeding. LL induced robust oscillations of adipose clock genes, including Nr1d1 and Dbp, under DRF (Figure 3B). LL facilitated complete phase inversion of Per2 and Arntl in VAT (Figure 3B). Clock genes in heart were phase inverted under DRF in LL-conditioned female mice (Figure 3B). Diurnal oscillations of Nr1d1, Dbp, and Per2 in kidney were phase inverted as well under LL + DRF; however, renal Arntl was only moderately phase shifted under LL + DRF (Figure S3E). Our results indicated that removal of the time cues from the central clock by LL promotes readily entrainment of extra-hepatic tissue clocks by feeding.

Overall, our data showed that cell-autonomous circadian clocks entrained to 7-day inverted feeding with different kinetics, which is gated by light-related cues.

### Inverted feeding reverses diurnal transcriptomes in VAT and liver

To determine phase entrainment of diurnal transcriptomes in peripheral tissues, we examined phase shifts of diurnal genes that oscillated under both tRF regimens (hereafter named as dual-oscillating genes). Considering the cyclic nature of circadian rhythms, absolute value of phase shift was presented. For example, a phase delay of 14 h is equal to a phase advance of 10 h (= 24 h–14 h) and would be presented as a phase shift of 10 h. Considering the phase resolution limited by the 4-h sampling interval, we defined phase shift within 4 h (0–4 h) as the phase-locked status and phase shift between 8 and 12 h as the phase-inverted status (8–12 h).

**Figure 4:**
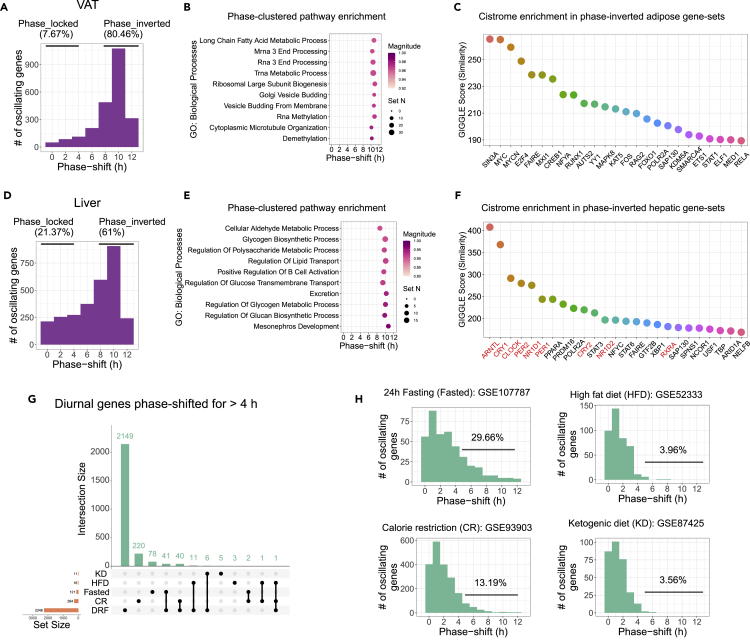
Inverted feeding reverses diurnal transcriptomes in VAT and liver (A) Phase-shift histogram shows that 80.46% of the adipose dual-oscillating genes were phase inverted by inverted feeding. Phase-locked, phase shift of 0–4 h; phase-inverted, phase shift of 8–12 h. Considering the cyclic nature, phase shift is converted to absolute value to allow a window of 0–12 h, e.g. a phase advance of 14 h equals to a phase delay of 10 h (24–14 = 10), and is expressed as a phase shift of 10 h. See also Figure S4A. VAT, visceral adipose tissue. (B) Top 10 phase-clustered pathways that are phase shifted by inverted feeding in VAT. Magnitude is a measure of the temporal cohesiveness of the phase set (q < 0.05). (C) Cistrome enrichment analysis of phase-inverted genes in VAT based on curated cistromes of transcription factors and chromatin regulators from CistromeDB. Circadian clock proteins are marked in red. See also Figure S4B. (D) Phase-shift histogram shows that 61% of the hepatic dual-oscillating genes were phase inverted by inverted feeding. See also Figure S4C. (E) Top 10 phase-clustered pathways that are phase shifted by inverted feeding in liver. (F) Cistrome enrichment analysis of phase-inverted genes in liver based on curated cistromes of transcription factors and chromatin regulators from CistromeDB. Circadian clock proteins are marked in red. See also Figure S4D. (G) Interaction of diurnal transcripts in liver that shows phase modulation by various dietary treatments. KD, ketogenic diet; HFD, high-fat diet; Fasted, 24-h fasting; CR, calorie restriction. Rhythmicity is statistically thresholded at meta2d_BH.Q < 0.05. The period is set to 24 h. (H) Phase-shift histogram shows that the degree of food scarcity is positively correlated with the degree of phase entrainment in hepatic diurnal transcriptomes. The degree of phase entrainment by diets is represented as the percentage of diurnal transcripts that were phase shifted by more than 4 h.

In VAT, phase-shift histogram showed that 80.46% of dual-oscillating genes were phase inverted under DRF in VAT (Figure 4A). Adipose phase-inverted genes from DRF and NRF exhibited clusters around ZT9 and ZT20-23 (Figure S4A). Phase set enrichment analysis (PSEA) of dual-oscillating genes using Gene Ontology (GO) terms based on phase shifts revealed that highly phase-clustered pathways exhibited at least a 10-h phase-shift and enriched biological progresses such as long-chain fatty acid metabolism, RNA modification, and Golgi vesicle trafficking (Figure 4B, Table S3). Cistrome matching analysis based on published genome-wide binding sites of transcription factors (TF) and chromatin regulators in VAT found chromatin-binding signatures for histone deacetylase-dependent corepressor SIN3A, MYC, etc. near phase-inverted genes (Figure 4C). Cistrome matching analysis predicted histone acetylase EP300, and circadian regulators, such as NR3C1 (glucocorticoid receptor), HDAC3, NR1D1, and ARNTL, etc. as potential transcriptional regulators of phase-locked genes in VAT (Figure S4B).

In liver, 61% of dual-oscillating genes were phase inverted under DRF (Figure 4D). Phase-inverted genes exhibited enrichment around ZT23-0 under DRF (Figure S4C). PSEA of dual-oscillating genes based on phase shifts identified signatures of metabolism of complex carbohydrates, transport of lipids, and glucose (Figure 4E, Table S3). These metabolic pathways showed 8–11 h of phase shifts, which is consistent with existing studies (Vollmers et al., 2009; Zhang et al., 2016). Cistrome matching analysis in liver showed that circadian clock TFs, e.g. BMAL1, CRY1, CLOCK, PER2, and NR1D1, were among the TFs bound near phase-inverted genes (Figure 4F). Cistrome matching analysis showed that in the gene-set of phase-locked genes, components of RNA polymerase II, such as POLR2A and POLR2B, were among top 3 predicted chromatin-bound TFs (Figure S4D).

To explore potential dietary components driving phase entrainment of diurnal transcriptomes in liver, we performed an integrated analysis of our hepatic dataset and existing datasets of hepatic diurnal transcriptomes modulated by high-fat diet (HFD, GSE52333), ketogenic diet (KD, GSE87425), calorie restriction (CR, GSE93903), and 24-h fasting (Fasted, GSE107787). Because phase entrainment is rare in these datasets, we relaxed the threshold to include diurnal genes with a phase shift >4 h. We retrieved 2,248 phase-shifted diurnal genes in DRF, 624 in CR, 121 in Fasted, 16 in HFD, and 11 in KD (Figure 4G). About 34% (41/121) of phase-shifted diurnal genes in Fasted liver were present in DRF-treated liver, whereas only 6.4% (40/624) in CR liver were present in DRF-treated liver (Figure 4G). Considering the percentage of phase-shifted diurnal genes in dual-oscillating genes as an indicator, we found that food scarcity is positively associated with the degree of phase entrainment in hepatic diurnal transcriptomes, such as 29.66% in Fasted, 13.19% in CR, 3.96% in HFD, and 3.56% in KD (Figure 4H). The results suggested that fasting might condition phase entrainment of hepatic diurnal transcriptome under light/dark cycles.

Next, we determined phase entrainment of hepatic diurnal lncRNA-omes by feeding. We uncovered 288 diurnal lncRNA gene-derived lncRNAs in liver. About 79.1% of the 66 dual-oscillating lncRNAs were phase inverted by inverted feeding (Figures S4E and S4F), suggesting that lncRNA profiles exhibit a faster kinetics than the total transcriptome (61%) in phase entrainment. Atrolnc-1 (1110038B12Rik) is recently shown to be induced by catabolic signals, including nutrient deprivation, and contributes to muscle wasting (Sun et al., 2018). Atrolnc-1 exhibited robust entrainment to inverted feeding in liver, based on mapped reads (Figure S4G). Data mining via CirGRDB showed that diurnal oscillation of Atrolnc-1 was enabled by calorie restriction in mouse liver (peak ZT9) (Li et al., 2018; Sato et al., 2017). We confirmed the robust phase entrainment of four hepatic lncRNAs, including Atrolnc-1 by RT-qPCR analysis (Figure S4H).

In sum, diurnal transcriptomes in metabolic tissues, such as VAT and liver, entrained readily to inverted feeding. Adipose diurnal transcriptomes showed higher completeness in phase entrainment by feeding than hepatic diurnal transcriptomes. Signatures of circadian clock TFs were enriched in phase-locked genes in VAT and phase-inverted genes in liver.

### Diurnal transcriptomes in kidney and heart show slow kinetics/resistance in phase entrainment to feeding

**Figure 5:**
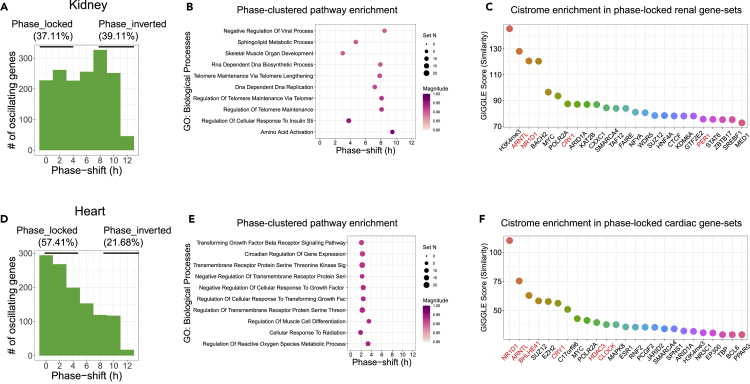
Diurnal transcriptomes in kidney and heart show slow kinetics/resistance in phase entrainment to feeding (A) Phase-shift histogram shows that 39.11% of the renal dual-oscillating genes were phase inverted by inverted feeding. Phase-locked, phase shift of 0–4 h; phase-inverted, phase shift of 8–12 h. Considering the cyclic nature, phase shift is converted to absolute value to allow a window of 0–12 h, e.g. a phase advance of 14 h equals to a phase delay of 10 h (24–14 = 10), and is expressed as a phase shift of 10 h. See also Figure S5A. (B) Top 10 phase-clustered pathways that are phase shifted by inverted feeding in kidney. Magnitude is a measure of the temporal cohesiveness of the phase set (q < 0.05). (C) Cistrome enrichment analysis of phase-locked genes in kidney based on curated cistromes of transcription factors and chromatin regulators from CistromeDB. Circadian clock proteins are marked in marked. See also Figure S5B. (D) Phase-shift histogram shows that 21.68% of the cardiac dual-oscillating genes were phase inverted by inverted feeding. See also Figure S5C. (E) Top 10 phase-clustered pathways that are phase shifted by inverted feeding in heart. (F) Cistrome enrichment analysis of phase-locked genes in heart based on curated cistromes of transcription factors and chromatin regulators from CistromeDB. Circadian clock proteins are marked in red. See also Figure S5D.

In kidney, 37.11% of the dual-oscillating genes remained phase locked to the light-dark cycle, whereas 39.11% were phase inverted (Figure 5A). Renal phase-inverted genes were clustered around ZT9 and ZT23 (Figure S5A). Pathway enrichment via PSEA revealed that phase-shift distribution of the ten most enriched pathways spanned from 4 h (cellular response to insulin stimulation), 6 h (telomere biology), to 9–10 h (amino acid activation) (Figure 5B, Table S3). Cistrome matching analysis showed that circadian TFs and histone H3 lysine 4 trimethylation (H3K4me3) were among top TFs and histone marks enriched in the vicinity of phase-locked genes (Figure 5C), whereas PPARA, FOXO1, etc. were among the top TFs bound to phase-inverted genes (Figure S5B).

In heart, the majority (57.41%) of dual-oscillating genes were phase locked to light-dark cycles, and only 21.68% of genes were phase inverted by inverted feeding (Figure 5D). Phase-inverted genes were clustered around ZT0 and ZT9, respectively (Figure S5C). PSEA revealed that almost all top 10 enriched pathways, including circadian clock, redox metabolism, and TGFβ signaling, remained phase locked to light/dark cycles (Figure 5E, Table S3). To explore regulatory mechanisms, we performed cistrome-matching analysis and found that circadian clock transcription factors, (e.g. NR1D1, ARNTL, BHLHE41, and CRY1) and components of polycomb repressive complex 2 (e.g. SUZ12 and EZH2) were top predicted TFs governing the phase-locked gene sets (Figure 5F), whereas FOXK1, FOXO1 and CREB1 were among top TFs bound to phase-inverted genes (Figure S5D). We tested the robustness of the chronotype in heart in an independent cohort (n = 1, sampled every 4 h for 24 h) and reproduced the findings on slow kinetics and/or resistance in phase entrainment by feeding (Figure S5E).

In sum, diurnal transcriptomes in kidney and heart showed slow kinetics and/or resistance in phase entrainment by inverted feeding. Cardiac diurnal transcriptomes exhibited lower completeness in phase entrainment than renal diurnal transcriptomes. Phase-locked genes in these tissues enriched signatures of circadian clock TFs.

### Phase entrainment of diurnal lipidomes and metabolomes in VAT and liver by inverted feeding

To match our findings in diurnal transcriptomes in metabolic tissues, we assessed phase entrainment of diurnal lipidomes in VAT and diurnal metabolomes in liver using targeted lipidomics and untargeted metabolomics, respectively.

**Figure 6:**
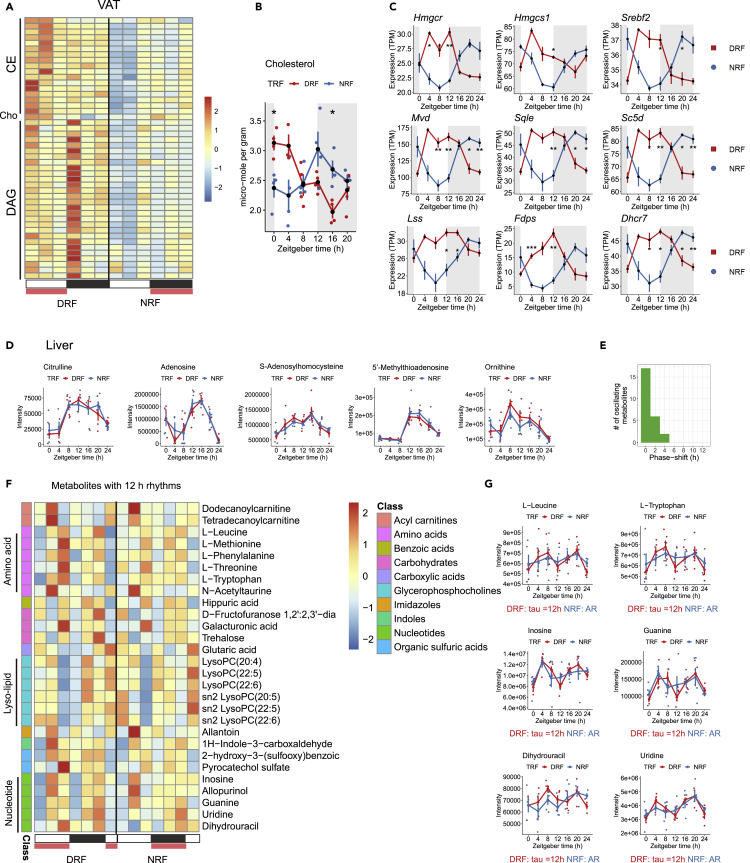
Phase entrainment of diurnal lipidomes and metabolomes in VAT and liver by inverted feeding (A) Heatmap showing 24 h profiles of neutral lipids by targeted lipidomics in VAT. Cho, cholesterol. See also Figures S6A and S6B. (B) Cholesterol rhythm is reversed by inverted feeding in VAT. Data were represented as mean ± sem (n = 4 except n = 3 in DRF ZT4 group). (C) Diurnal expression of genes in cholesterol biosynthesis process is reversed by inverted feeding in VAT. See also Figure S6C. (D) Twenty-four-hour profiles of representative diurnal metabolites in liver under tRF regimens. See also Figure S6D. (E) Phase-shift diagram of dual-oscillating metabolites in liver. (F) Heatmap showing 24 h of hepatic metabolites with 12-h rhythms. These metabolites oscillated under at least one tRF regimen. (G) Twenty-four-hour profiles of representative hepatic metabolites with 12-h rhythms under tRF regimens. Tau, the period length. Data were represented as mean ± sem (n = 4–5 for metabolites, n = 4 for genes). Multiple t tests with Bonferroni correction; p *≥* 0.05 is not shown, ∗p < 0.05, ∗∗p < 0.01, ∗∗∗p < 0.001.

In VAT, targeted lipidomics profiling of 199 neutral lipids showed that only five species oscillated in a diurnal manner under tRF regimens, which are composed of free cholesterol (Cho) and some cholesterol esters (CE) (Figure 6A, Table S4). Cholesterol rhythm was robust under DRF (Figure 6B, p < 0.0001, phase 2 h). Apparently, cholesterol rhythm under NRF reached the peak around 12 h. PSEA and diurnal expression profiles of cholesterol biosynthetic genes revealed that cholesterol biosynthetic process was significantly enriched (q-value 0, magnitude 0.85) and phase shifted by 10 h (Figures 6C, Table S3). We noticed and confirmed by MetaCycle rhythmicity analysis that diacylglycerol (DAG) species exhibited a 12-h oscillation under DRF (Figure S6A, Table S4). Triacylglycerol (TAG) species were not oscillating in adipose tissue but showed high levels at ZT8 under DRF (Figure S6B). By integrating with the gene panel on triglyceride metabolism, the alterations on daily profiles of DAG/TAG were associated with phase inversion of Dgat1 and Lipe (HSL) rhythms (Figure S6C). Thus, our integrated analysis of adipose lipidomes and transcriptomes showed that cholesterol rhythm entrained readily to inverted feeding, and DAG levels were induced to oscillate in a 12-h manner under DRF in VAT.

In liver, untargeted metabolomics analysis uncovered diurnal rhythms in 68 out of 243 metabolites in liver (Figure S6D). These diurnal metabolites include robust circadian metabolites in liver, e.g. citrulline, adenosine, S-adenosylhomocysteine (SAH), 5′-methylthioadenosine, and ornithine (Figure 6D). All of the 25 dual-oscillating metabolites remained phase locked to light-dark cycle (Figure 6E, Table S4). However, we detected 28 metabolites that oscillated in a 12-h rhythm, which include amino acids, lyso-lipids, and nucleotide/nucleosides (Figure 6F). Apparently, metabolites involved in amino acid metabolism and nucleotide metabolism oscillated only under DRF (Figure 6F, Table S4), such as L-leucine, L-tryptophan, inosine, guanine, dihydrouracil, and uridine (Figure 6G). Metabolites related to lyso-lipids oscillated only under NRF (Table S4). This metabolic signature suggested that diurnal rhythms of some metabolic pathways in liver were not entrained by feed-fast cycle. Instead, inverted feeding licensed the 12-h oscillations of metabolites related to amino acids and nucleotides in liver.

### Inverted feeding entrains diurnal rhythms of fatty acid oxidation in heart

**Figure 7:**
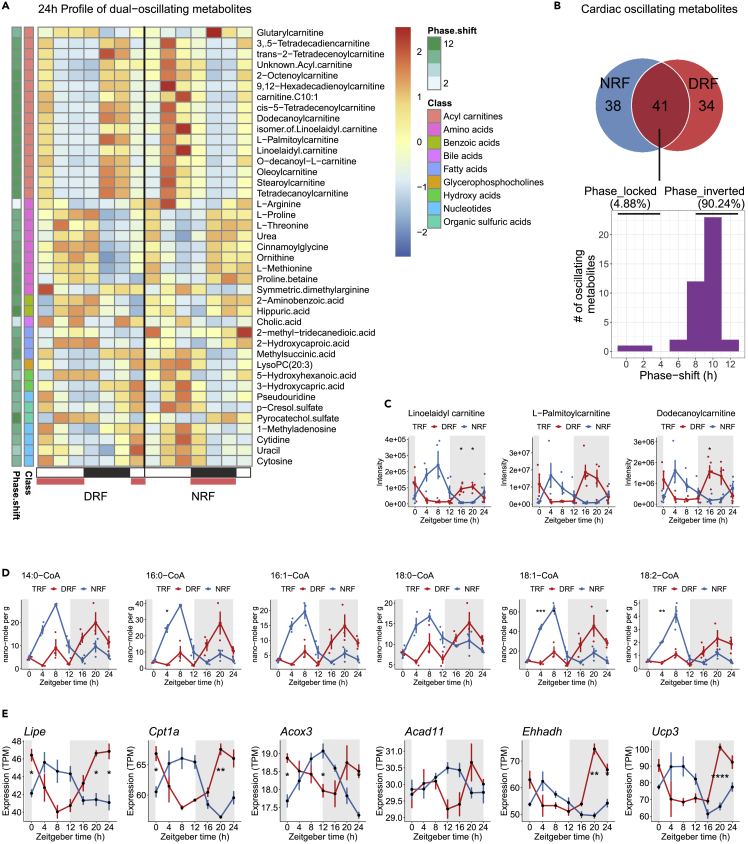
Inverted feeding entrains diurnal rhythms of fatty acid oxidation in heart (A) Heatmap showing 24-h profiles of all dual-oscillating metabolites in heart. Intensity levels are represented as the Z score scaled means. See Figure S7A. (B) Venn diagram and phase-shift diagram showing the interaction between diurnal metabolites in heart under tRF regimens, 90.24% of which were phase inverted. (C) Representative 24-h profiles of acylcarnitines in heart. (D) Twenty-four-hour targeted lipidomics profiles of long-chain acyl-CoAs in heart. (E) Twenty-four-hour expression profiles of diurnal genes involved in fatty acid oxidation. Red, DRF; blue, NRF. Data were represented as mean ± sem (n = 4 for genes, n = 4–5 for metabolites, n = 3 for acyl-CoAs). Multiple t tests with Bonferroni correction; p *≥* 0.05 is not shown, ∗p < 0.05, ∗∗p < 0.01, ∗∗∗p < 0.001.

To determine how inverted feeding entrains diurnal physiology and metabolism in heart, we integrated diurnal transcriptomes and metabolomes in heart tissue. Untargeted metabolomics analysis of diurnal metabolites in heart uncovered 113 diurnal metabolites (Figure S7, Table S4). Clustering analysis based on diurnal profiles of these metabolites under both tRF regimens produced six clusters of metabolites. Particularly, cluster 3 metabolites are mainly composed of acylcarnitines and some metabolites in nucleotide metabolism and energy metabolism (Figure S7). We further found that inverted feeding reversed diurnal rhythms of the 41 dual-oscillating diurnal metabolites in heart tissue, including three major classes, such as acylcarnitine, amino acid, and nucleotide (Figure 7A). Phase-inverted metabolites accounted for 90.24% of the dual-oscillating metabolites (Figure 7B). Thus, despite the slow kinetics and/or resistance in phase entrainment of cardiac transcriptomes by feeding, inverted feeding entrained diurnal metabolomes in heart readily within one week.

Fatty acid oxidation is key to maintain energy metabolism and tissue homeostasis in heart. As shown in Figure 7C, the peak time of cardiac acylcarnitine species fell in the inactive/food-restricted period, reflecting the priority of fat burning in the postprandial phase in heart tissue. Targeted lipidomics profiling of acyl-CoA species showed that inverted feeding reversed the phase of cardiac acyl-CoA species in synchrony with its actions on acylcarnitines (Figure 7D, Table S4). These coordinated entrainment of substrates and intermediates of fatty acid oxidation by inverted feeding is associated with transcriptional changes. Diurnal rhythms of major genes involved in lipolysis and fatty acid oxidation, such as Lipe, Cpt1a, Acox3, Acad11, Ehhadh, and Ucp3, were phase inverted by inverted feeding in heart (Figure 7E).

In summary, diurnal metabolome of the heart is readily responsive to phase entrainment by feeding, particularly regarding metabolites involved in fatty acid oxidation. This metabolic reprogramming by feeding time is coordinated with fast kinetics in entraining transcriptional rhythms of fatty acid oxidative genes to feed-fast cycles in heart.

## Discussion

We took a multi-omics approach to study entrainment of diurnal rhythms and circadian clocks by inverted feeding in mouse peripheral tissues. Diurnal transcriptomics revealed tissue-specific kinetics in phase entrainment by feeding. Metabolic tissues, such as fat tissue (80.46% of dual-oscillating genes) and liver (61%) entrained readily to inverted feeding, whereas kidney (39.11%) and heart (21.68%) exhibited less completeness in food entrainment within one week of inverted feeding. We further showed that light-related cues condition phase entrainment of clocks by feeding in extra-hepatic peripheral tissues. To explore the impact on physiology and metabolism, we performed integrated analysis of metabolites, lipids, and transcripts and found that cholesterol rhythm entrained readily to inverted feeding in fat tissue and that diurnal rhythms of fatty acid oxidation entrained almost completely to inverted feeding in heart, both of which occurred with coordinated entrainment of metabolic gene transcripts.

In the past, much has been focused on entrainment of the liver clock by feeding. Nutrient-sensing pathways, such as glucose-sensing AMPK/OGT, NAD^+^-sensing sirtuins/PARP1, and PGC-1α, couple circadian clocks to nutrient availability (Kim and Lazar, 2020; Reinke and Asher, 2019). Feeding rhythms accounted for 84% of phase-shifted transcripts in mouse liver under constant darkness (Vollmers et al., 2009). Early studies suggested that tissue clocks might entrain to feeding rhythms with different speeds (Damiola et al., 2000; Stokkan et al., 2001). Recently, Wang et al. showed that tRF shifted the phase of the skin clock by 3–4 h (Wang et al., 2017). In our study, we found distinct kinetics in entrainment of diurnal transcriptomes and tissue clocks by feeding. Metabolic tissues exhibited faster kinetics, whereas heart and kidney had slower kinetics. This effect tracks with phase kinetics of tissue clocks. These findings suggested that the connection between clock and metabolism is highly tissue specific. It is tempting to speculate that the desynchrony of diurnal transcriptomes among peripheral tissues under irregular eating time may contribute to the pathogenesis of metabolic diseases, in addition to the well-recognized desynchrony between the SCN clock and peripheral clocks (Longo and Panda, 2016; Rubino et al., 2020).

Feed-fast cycles provide a dominant synchronizing signal to reset the liver clock. Recent studies suggested that peripheral clocks in liver and skin were modulated by light-dependent signaling and clocks in other tissues (Koronowski et al., 2019; Ray et al., 2020; Welz et al., 2019). Our results are consistent with these findings and show that light-related cues gate clock entrainment in peripheral tissues. Particularly, removal of time cues from the SCN clock by LL either consolidates or facilitates phase entrainment by feeding in extrahepatic tissues. The identity of light-related signaling under tRF remains to be characterized. It has been shown that the hypothalamus-pituitary-adrenal axis, sympathetic tones, and body temperature-sensitive signaling entrain the liver clock from the light-responsive SCN clock (Liu et al., 2019; Schibler et al., 2016). Thus, a tissue-specific balancing act between light- and food-induced signaling pathways may determine the kinetics in entrainment of peripheral clocks and diurnal rhythms by feeding.

Female mice have not been widely used in tRF studies. Our study used female mice for multi-omics profiling and compared entrainment of peripheral clocks between females and males. We observed sex-related difference in the robustness of peripheral clocks under DRF, particularly in heart and kidney. It has been recognized recently that behavioral rhythms are more consolidated in females than in males (Anderson and FitzGerald, 2020). Weger et al. showed that not all but about 71% diurnal transcripts and 55% diurnal metabolites in liver are conserved between male and female mice, which is conditioned by microbiome (Weger et al., 2019). Thus, this resource could serve as a primer to explore mechanisms underneath sexual dimorphism in food entrainment, which may have implications for susceptibility of males to metabolic diseases.

The landscape of circadian physiology and metabolism entrained by feeding is key to understand the mechanisms and health benefits of tRF. In heart, we found coordinated phase entrainment of fatty acid metabolic genes and acylcarnitine metabolites. It is well established that the myocardial circadian clock regulates diurnal rhythms of mitochondrial oxidative metabolism and fatty acid utilization in heart, which is essential for longevity and health span (Zhang et al., 2020a). Clock-regulated transcription factor KLF15 conditions diurnal rhythms of susceptibility to heart injury, in part through fatty acid metabolism and electrophysiology (Jeyaraj et al., 2012; Li et al., 2020; Zhang et al., 2015a). These findings corroborated the notion that the circadian coherence of energy metabolism in heart is shaped by feed-fast cycles and circadian clocks. Because circadian reprogramming of fatty acid metabolism occurs prior to transcriptional changes of the circadian clock, it is likely that post-transcriptional regulation of fatty acid metabolism, possibly at the levels of proteins and activity, may account for diurnal rhythms of cardiac energy metabolism under tRF. In that sense, circadian profiling of proteomes and/or proteomes of a specific post-translational modification in heart would provide mechanistic insights in the future.

In adipose, we found robust phase entrainment of free cholesterol in synchrony with genes in cholesterol biosynthetic process. It is perplexing because VAT is not a major organ for cholesterol biosynthesis (Luo et al., 2020). The apparent clock-independent phase entrainment in VAT suggested that fat tissue might utilize an unconventional mechanism during food entrainment, which may recruit non-clock TFs such as nuclear receptors and sterol response element-binding proteins as suggested in recent studies (Eckel-Mahan et al., 2013; Guan et al., 2018; Yang et al., 2006; Zhang et al., 2015b). In liver, we did not find any diurnal metabolite that had entrained to inverted feeding; however, we found robust 12-h rhythms in many amino acids and nucleotides, including leucine. As an activator of mTOR signaling, this 12-h tone of leucine may orchestrate diurnal rhythms of metabolism and physiology via protein phosphorylation as suggested recently (Robles et al., 2017). In addition, signaling metabolites with 24 h or 12 h rhythms modulated by DRF may act not only in local tissues but also in dialogue between tissues (Dyar et al., 2018; Koronowski and Sassone-Corsi, 2021). Thus, this multi-tissue resource would facilitate the community to explore the functions of tissue-specific signature transcripts and metabolites connecting feed-fast cycles and circadian biology.

### Limitations of study

Our study has several limitations. First, we have only examined four peripheral tissues. Inclusion of additional organs in other physiological systems, such as respiratory system, gastrointestinal system, endocrine system, nervous system, and musculoskeletal system, would be necessary to explore system-wide kinetics and regulatory mechanisms in entrainment of circadian biology by feeding. Second, transcriptomics and metabolomics are merely snap-shots of transcription and metabolism. To uncover the dynamics and kinetics, applications such as Global Run-On sequencing (GRO-seq) and metabolic flux analysis would be required.

### Resource availability

#### Lead contact

Further information and requests for resources and reagents should be directed to and will be fulfilled by the lead contact, Min-Dian Li (mindianli@tmmu.edu.cn).

#### Materials availability

This study did not generate new reagents.

#### Data and code availability

Original/source data and code for metabolomics and Figures in the paper are available [i.e., Mendeley Data https://doi.org/10.17632/mb25x9t4m7.1]. The accession numbers for the transcriptomics data reported in this paper are NCBI GEO: GSE150380, GSE150381, GSE151221, GSE151228, and CNGBdb: CNP0001605, CNP0001638, CNP0001639, CNP0001640. CircaMet database: https://www.CircaMetDB.org.cn.

## Methods

All methods can be found in the accompanying transparent methods supplemental file.

## Supplemental information

Document S1. Transparent methods, Figures S1–S7, and Table S6

Table S1. Summary statistics of RNA-seq, related to Figure 1 This Table summarizes the volume of raw reads (millions), clean reads (millions), clean bases (gigabytes) and sequencing depth (millions), Quality score (Q20 and Q30). Tissue types include heart, liver, kidney, and VAT. Sample name refers to the type of time-restricted feeding (D/N), Zeitgeber time (00, 04, 08, 12, 16, 20, 24 h), and biological replicate assignment (_1, _2, _3, _4).

Table S2. Oscillating genes and LncRNAs, related to Figures 1, 4, and 5 This table itemizes rhythmicity of oscillating genes and long non-coding RNAs (lncRNA). MetaCycle-imputed rhythmicity parameters contain p-value, FDR value (BH.Q), period, phase, basal level (Base), amplitude (AMP), and relative amplitude (rAMP) values. CycID, Ensembl ID (if not available, use Gene Symbol); gene_name, Gene Symbol; gene_locus or transcript_locus, chromosomal location of a gene or transcript; tRF, time-restricted feeding; DRF, daytime-restricted feeding; NRF, nighttime-restricted feeding; gene_biotype, gene or transcript classification.

Table S3. Results of phase set enrichment analysis, related to Figures 4 and 5 This table lists results of phase set enrichment analysis of dual-oscillating genes in peripheral tissues.

Table S4. Oscillating metabolites and lipids, related to Figures 6 and 7 This table itemizes rhythmicity of oscillating metabolites in liver and heart, neutral lipids in VAT, and all acyl coenzyme A species in heart. MetaCycle-imputed rhythmicity parameters contain p-value, FDR value (BH.Q), period, phase, basal level (Base), amplitude (AMP), and relative amplitude (rAMP) values. tRF, time-restricted feeding; DRF, daytime-restricted feeding; NRF, nighttime-restricted feeding.

Table S5. Raw data for metabolomics and Lipidomics, related to Figures 6 and 7
