# Supplementary material for: A multi-tissue multi-omics analysis reveals distinct kineztics in entrainment of diurnal transcriptomes by inverted feeding
Source: iScience. 2021 Mar 19;24(4):102335. doi: 10.1016/j.isci.2021.102335 (PMC8050734; doi:10.1016/j.isci.2021.102335)
Supplement: Document S1. Transparent methods, Figures S1–S7, and Table S6 [file mmc1.pdf]

iScience, Volume *24*

Supplemental information

A multi-tissue multi-omics analysis reveals distinct kineztics in entrainment of diurnal transcriptomes by inverted feeding

Haoran Xin, Fang Deng, Meiyu Zhou, Rongfeng Huang, Xiaogen Ma, He Tian, Yan Tan, Xinghua Chen, Dan Deng, Guanghou Shui, Zhihui Zhang, and Min-Dian Li

Supplemental Documents

Supplemental Figure 1

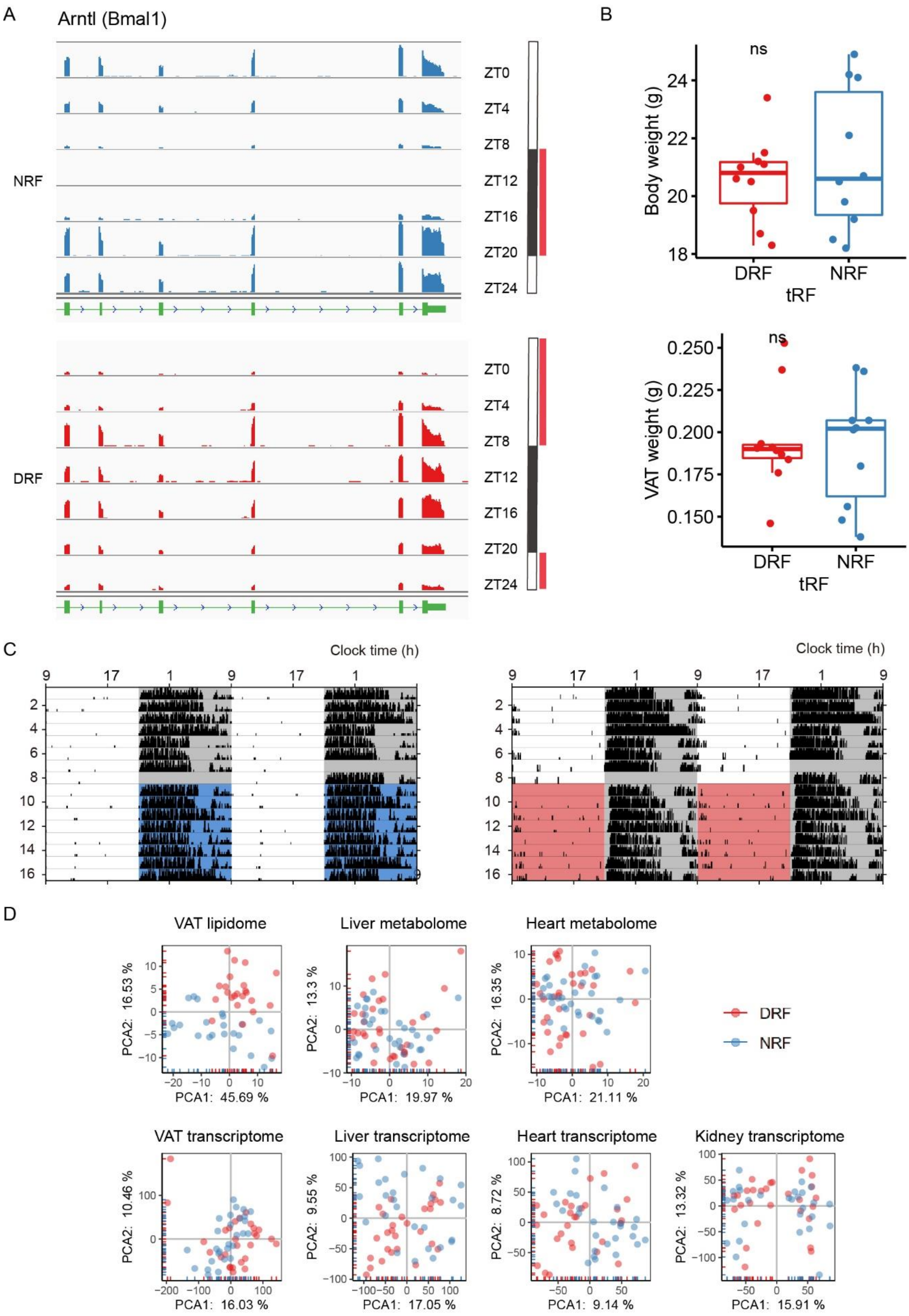

Figure S1, Related to Figure 1.

(A) Read counts from the clock output gene *Arntl* locus exhibit diurnal rhythms in liver.

(B) Records of diurnal body weight and visceral adipose tissue (VAT) weight of 9 weeks old female mice that had been on DRF or NRF for 1 week (n = 10). Data are represented in boxplots as a five-number summary, i.e. minimum, first quartile, median, third quartile, and maximum. Wilcoxon tests, $P >= 0.05$, ns, not significant.

(C) Wheel-running activity recordings of female mice under NRF (blue shade) and DRF (red shade). Blue/red shades indicate time of feeding. Horizontal axis indicates clock time in 24 hours. Vertical axis indicates days after the recording began. Representative actograms are shown.

(D) Principal component analysis of diurnal transcriptomes and metabolomes in peripheral tissues subjected to tRF regimens. Eigen value is indicated after PCA1 and PCA2.

Supplemental Figure 2

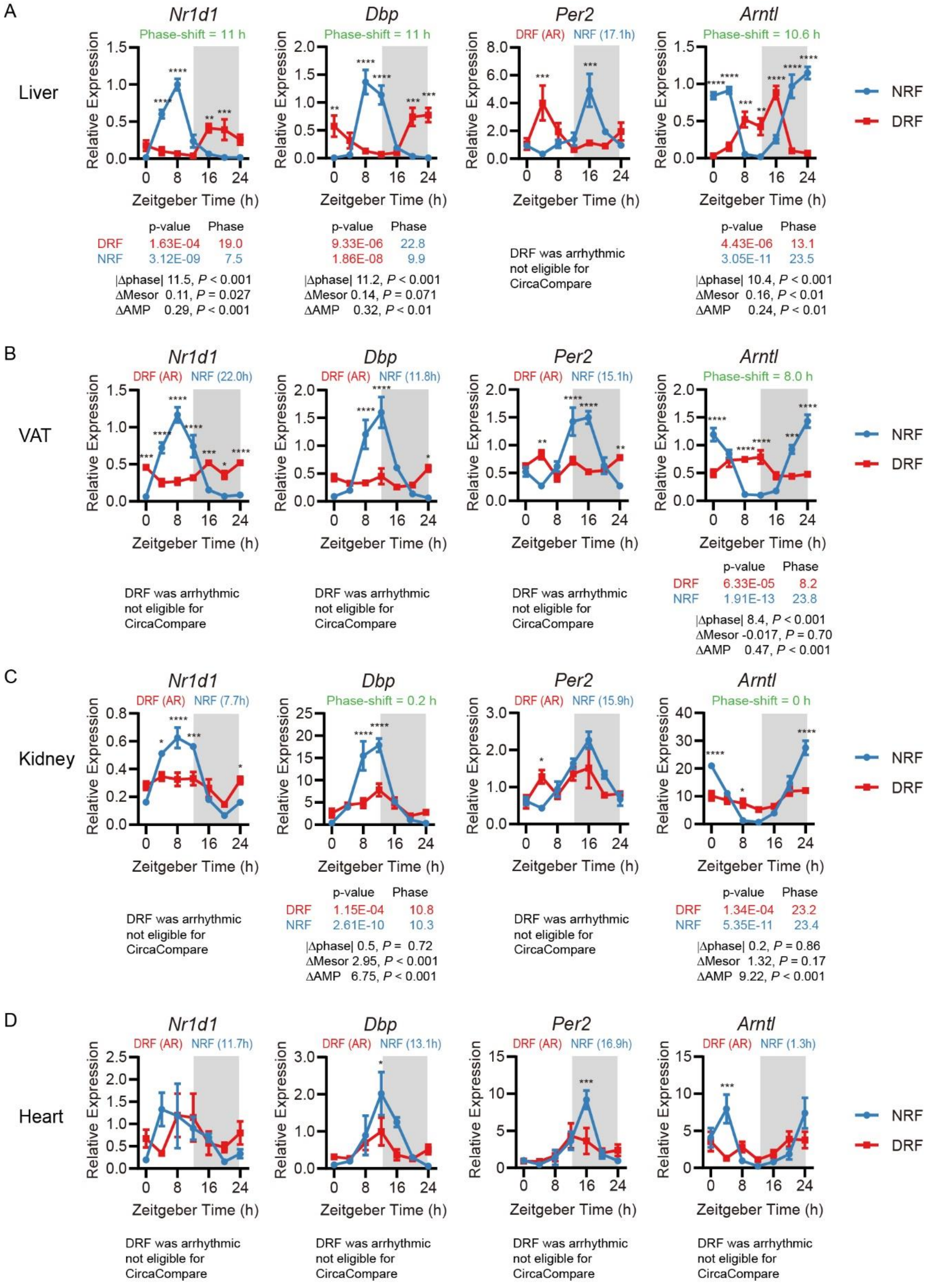

Figure S2. Clock Entrainment by Inverted Feeding in Male Mice, Related to Figure 2. (A-D) Diurnal expression of clock genes in liver (**A**), VAT (**B**), kidney (**C**), and heart (**D**). C57BL/6J male mice at the age of 7 weeks were acclimated to 12/12h light/dark cycle for one

week, and subjected to tRF regimens for 7 days. Data are represented as mean ± sem (n = 4, except n = 3 in kidney ZT0 group). Multiple t-tests with Bonferroni correction; not shown when *P* >= 0.05, **P* < 0.05, ** *P* < 0.01, *** *P* < 0.001. Circadian rhythmicity parameters were determined by MetaCycle (presented at the top of graphs) and CircaCompare (presented at the bottom of graphs) with the period set to 24 h. Phase shift is represented in absolute value. AR, arrhythmic; mesor, rhythm-adjusted mean expression level; AMP, amplitude.

Supplemental Figure 3

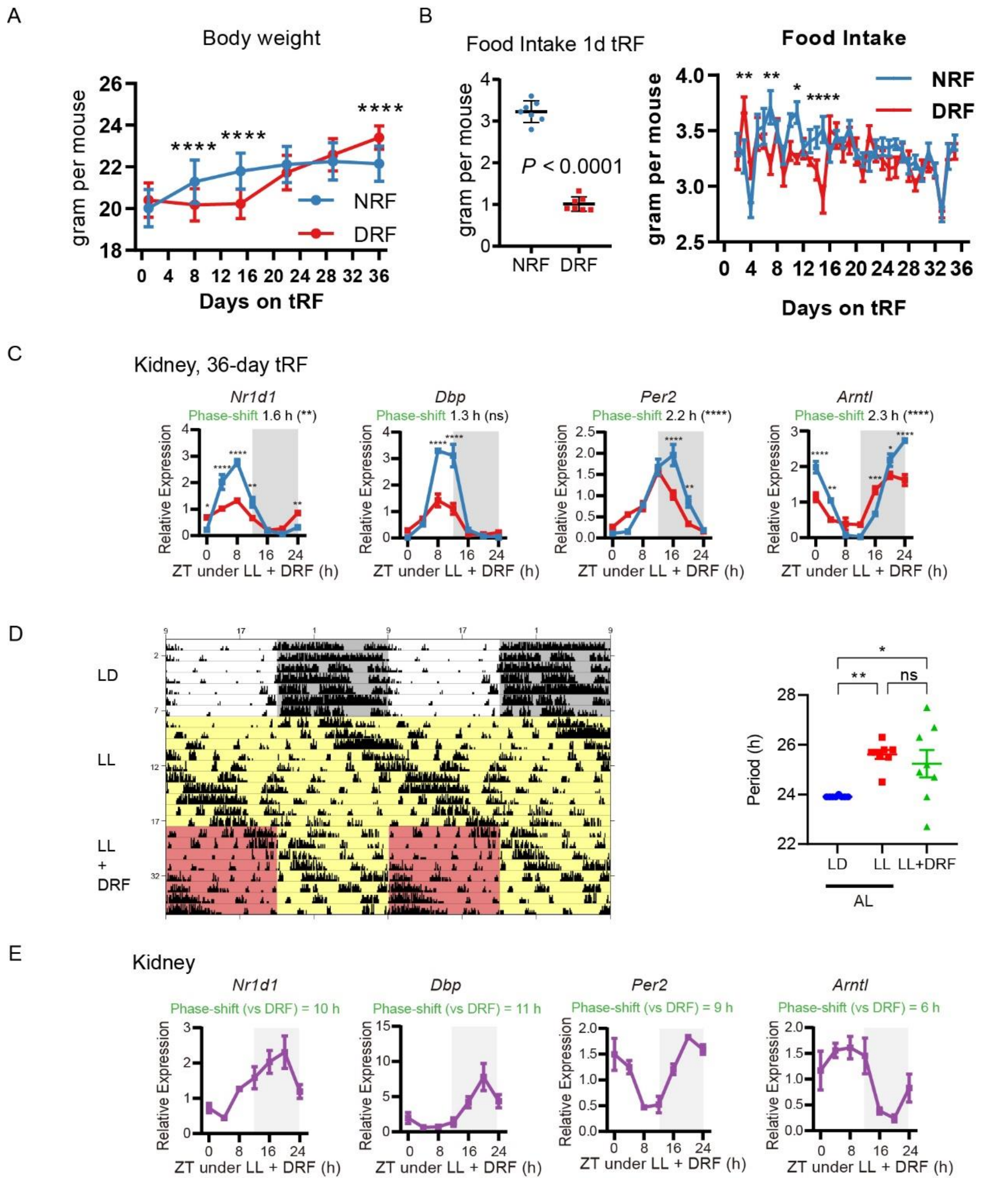

Figure S3. Effects of Duration and Constant Light on Clock Entrainment by Inverted Feeding, Related to Figure 3.

(A) Weekly monitoring of body weight from female mice on DRF or NRF. Data are represented as mean ± sd (n = 28). Multiple t-tests with Bonferroni correction; not shown when *P* >= 0.05, **** *P* < 0.0001.

(B) Daily monitoring of food intake from female mice on DRF or NRF. Data are represented as mean ± sd (n = 28 in total, average of food consumption from 7 cages containing 4 mice) for Day 1 and mean ± sem for other time-points. 1d tRF, Day 1 on time-restricted feeding. Student’s t-test or multiple t-tests with Bonferroni correction; not shown when *P* >= 0.05, *P < 0.05, ** *P* < 0.01, *** *P* < 0.001, **** *P* < 0.0001.

(C) Effects of 36-day inverted feeding on diurnal expression of clock genes in kidney. Phase-shifts were computed by CircaCompare. t-tests provided by CircaCompare or multiple t-tests with Bonferroni correction; ns or not shown when $P >= 0.05$, *$P < 0.05$, ** $P < 0.01$, *** $P < 0.001$, **** $P < 0.0001$.

(D) Wheel-running activity recordings and statistics of female mice under normal light/dark cycle (LD), constant light (LL, yellow shade) and LL+DRF (red/yellow shade). Red indicates feeding time. Horizontal axis indicates clock time in 24 hours. Vertical axis indicates days after the recording began. A representative actogram is shown. Data are represented as mean ± sem (n = 8). 1-way ANOVA with Bonferroni correction; ns, not significant when $P >= 0.05$, *$P < 0.05$, ** $P < 0.01$.

(E) Effects of constant light (LL) on diurnal expression of clock genes in kidney under DRF. Female mice were acclimated to 12h light-dark cycles, subjected to LL for 9 days, and then subjected to DRF and constant lightness for 7 days. Phase shift was estimated by meta2d_phase (h) with the period length under LL set to 20-28h and that under LD set to 24h. CircaCompare could not be applied because the period length under LL differs significantly from that under 12h light-dark cycles. DRF, daytime-restricted feeding; NRF, nighttime-restricted feeding. Data are represented as mean ± sem (n = 4). Multiple t-tests with Bonferroni correction; not shown when $P >= 0.05$, *$P < 0.05$, ** $P < 0.01$, *** $P < 0.001$.

Supplemental Figure 4

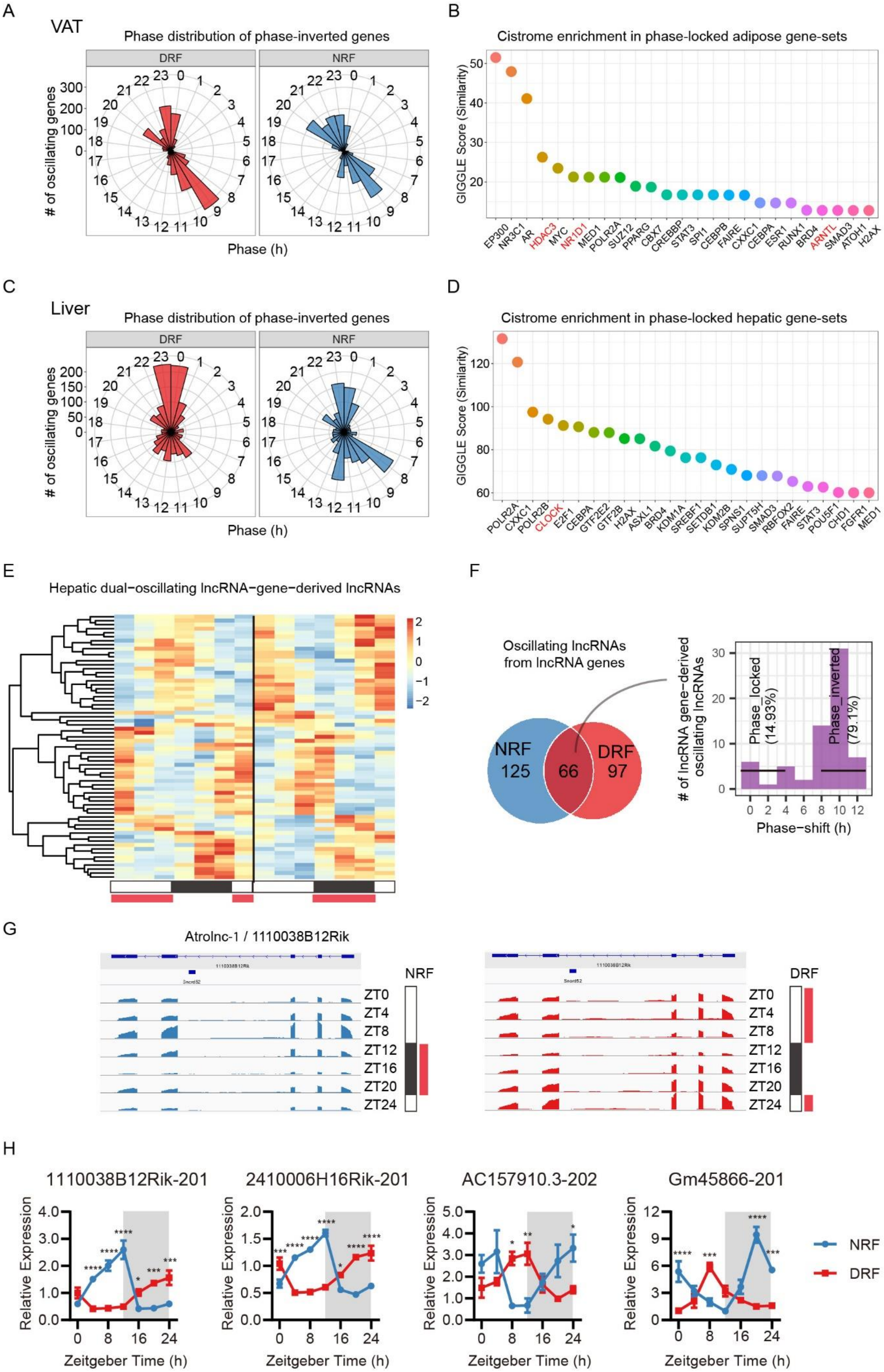

Figure S4. Circadian and Phase Analysis of Diurnal Transcriptomes in VAT and Liver, Related to Figure 4.

(A) Phase distribution of phase-inverted genes in VAT.

(B) Cistrome enrichment analysis of phase-locked genes in VAT based on curated hepatic cistromes of transcription factors and chromatin regulators from CistromeDB.

(C) Phase distribution of phase-inverted genes in liver.

(D) Cistrome enrichment analysis of phase-locked genes in liver based on curated hepatic cistromes of transcription factors and chromatin regulators from CistromeDB.

(E) Heatmap showing 24 h expression profiles of dual-oscillating lncRNA-gene-derived lncRNA transcripts in liver.

(F) 79.1% of the 66 oscillating lncRNAs from lncRNA genes in liver are phase-inverted by inverted feeding.

(G) Diurnal profiles of read counts of 1110038B12Bik (Atrolnc-1) lncRNA.

(F) 24h expression profiles of selected dual-oscillating lncRNA gene-derived lncRNAs. Data are represented as mean ± sem (n = 4). Multiple t-tests with Bonferroni correction; not shown when $P >= 0.05$, *$P < 0.05$, ** $P < 0.01$, *** $P < 0.001$, **** $P < 0.0001$.

Supplemental Figure 5

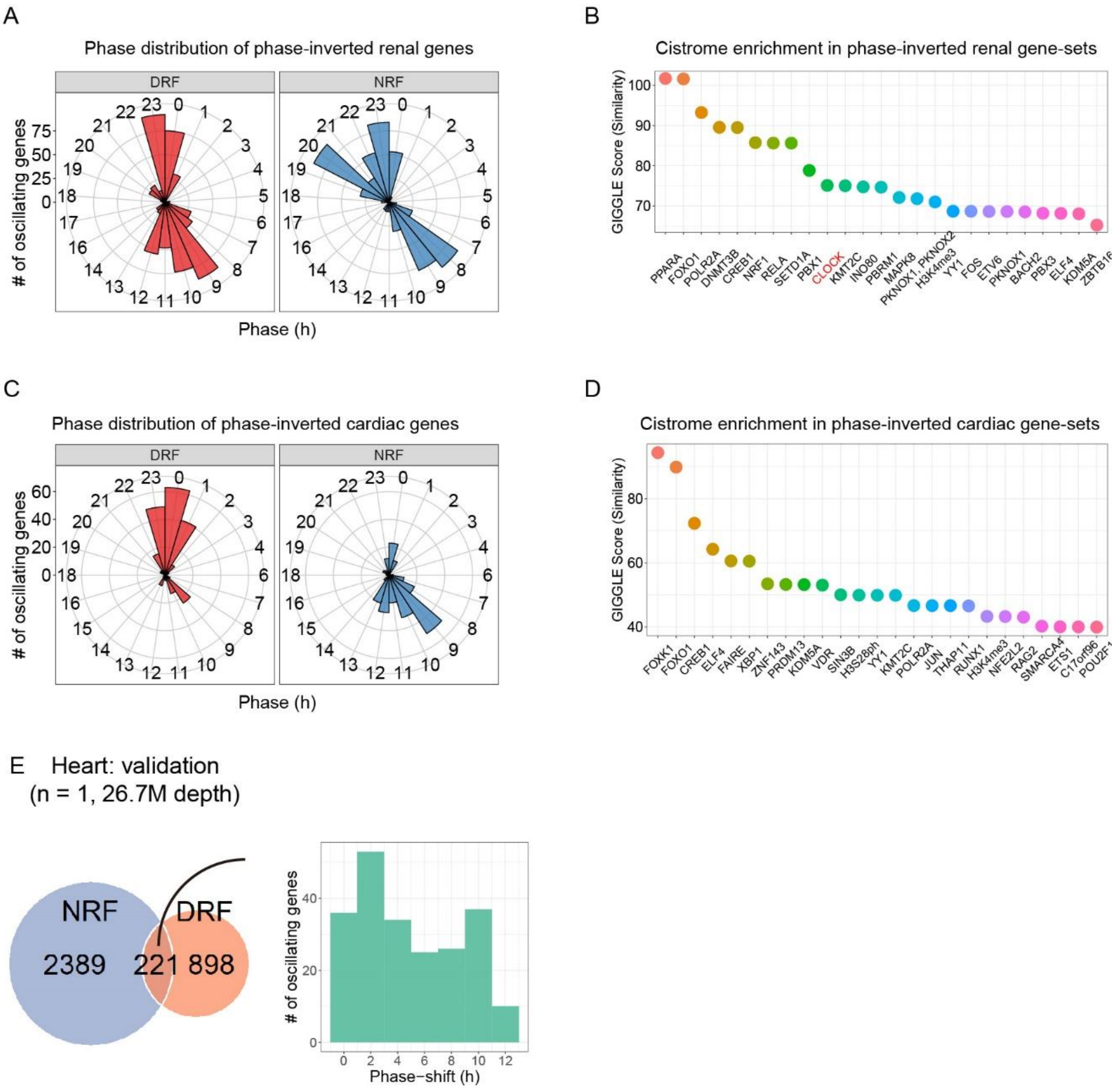

Figure S5. Circadian and Phase Analysis of Diurnal Transcriptomes in Kidney and Heart, Related to Figure 5.

(A) Phase distribution of phase-inverted genes in kidney.

(B) Cistrome enrichment analysis of phase-inverted genes in kidney based on curated hepatic cistromes of transcription factors and chromatin regulators from CistromeDB.

(C) Phase distribution of phase-inverted genes in heart.

(D) Cistrome enrichment analysis of phase-inverted genes in heart based on curated hepatic cistromes of transcription factors and chromatin regulators from CistromeDB.

(E) General feature of a validation cohort testing phase entrainment of cardiac diurnal transcriptome by feeding. RNA-seq data depth is at least 26.7 million reads. Rhythmicity is defined as meta2d_pvalue < 0.1, period is set to 24 h.

Supplemental Figure 6

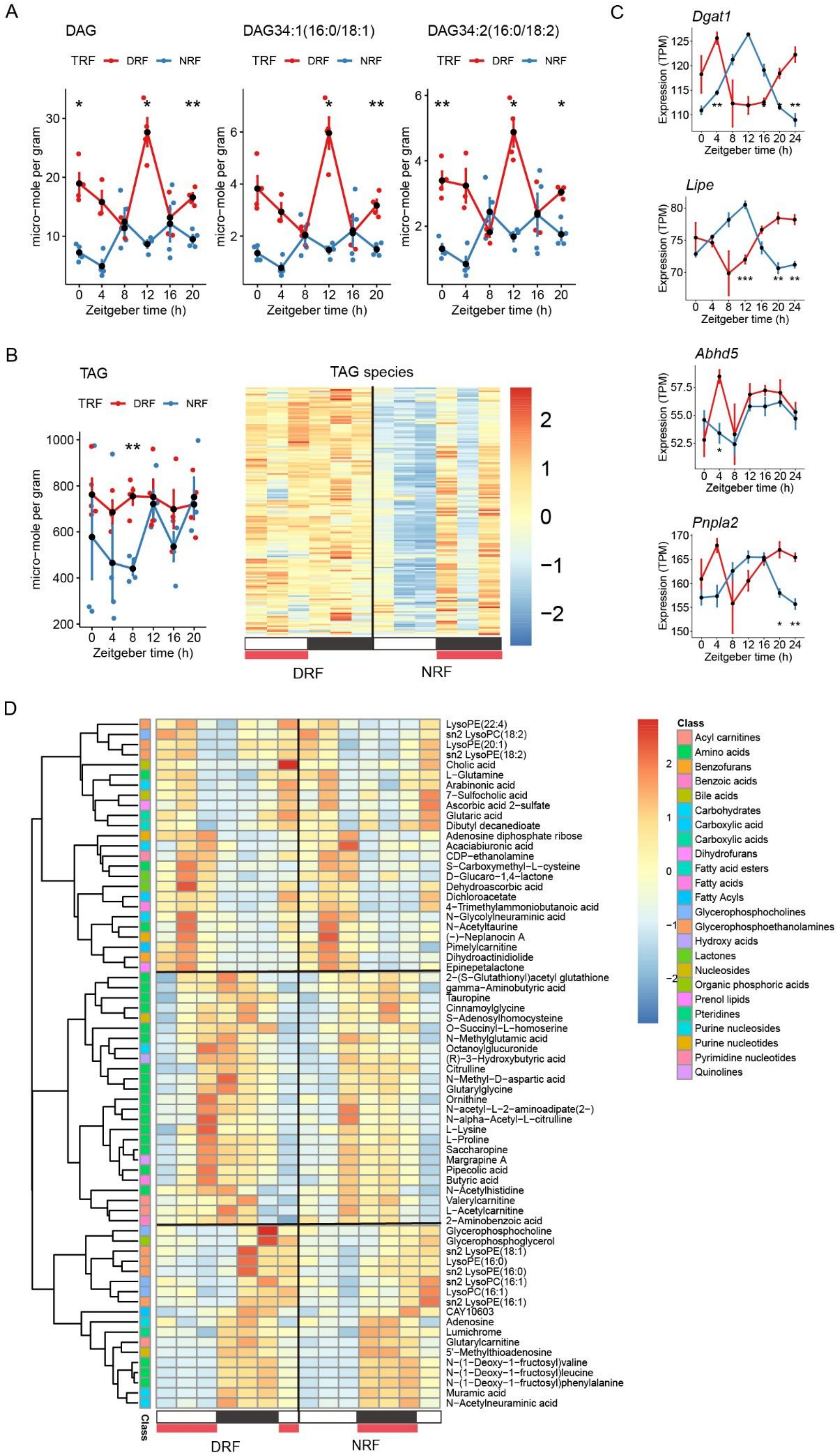

Figure S6. Circadian and Phase Analysis of Adipose Lipidome and Hepatic Metabolome, Related to Figure 6.

(A) Diurnal profiles of major diacylglycerol (DAG) species in adipose tissue. Data were represented as mean ± sem (n = 4 except n = 3 in DRF ZT4 group). Multiple t-tests with Bonferroni correction; $P >= 0.05$ is not shown; $^{*}P < 0.05$; $^{**}P < 0.01$.

(B) Heatmap showing diurnal profiles of triacylglycerols (TAG) in adipose tissue. ROUT outlier analysis was performed for total TAG levels, but no outlier was identified at a threshold Q of 0.01. Data were represented as mean ± sem (n = 4 except n = 3 in DRF ZT4 group). Multiple t-tests with Bonferroni correction; $P >= 0.05$ is not shown; $^{**}P < 0.01$.

(C) Diurnal expression profiles of genes involved in DAG/TAG metabolism. Data were represented as mean ± sem (n = 4). Multiple t-tests with Bonferroni correction; $P >= 0.05$ is not shown; $^{*}P < 0.05$; $^{**}P < 0.01$, $^{***}P < 0.001$.

(D) 24 h profiles of hepatic metabolites that oscillate under at least one tRF regimen.

Supplemental Figure 7

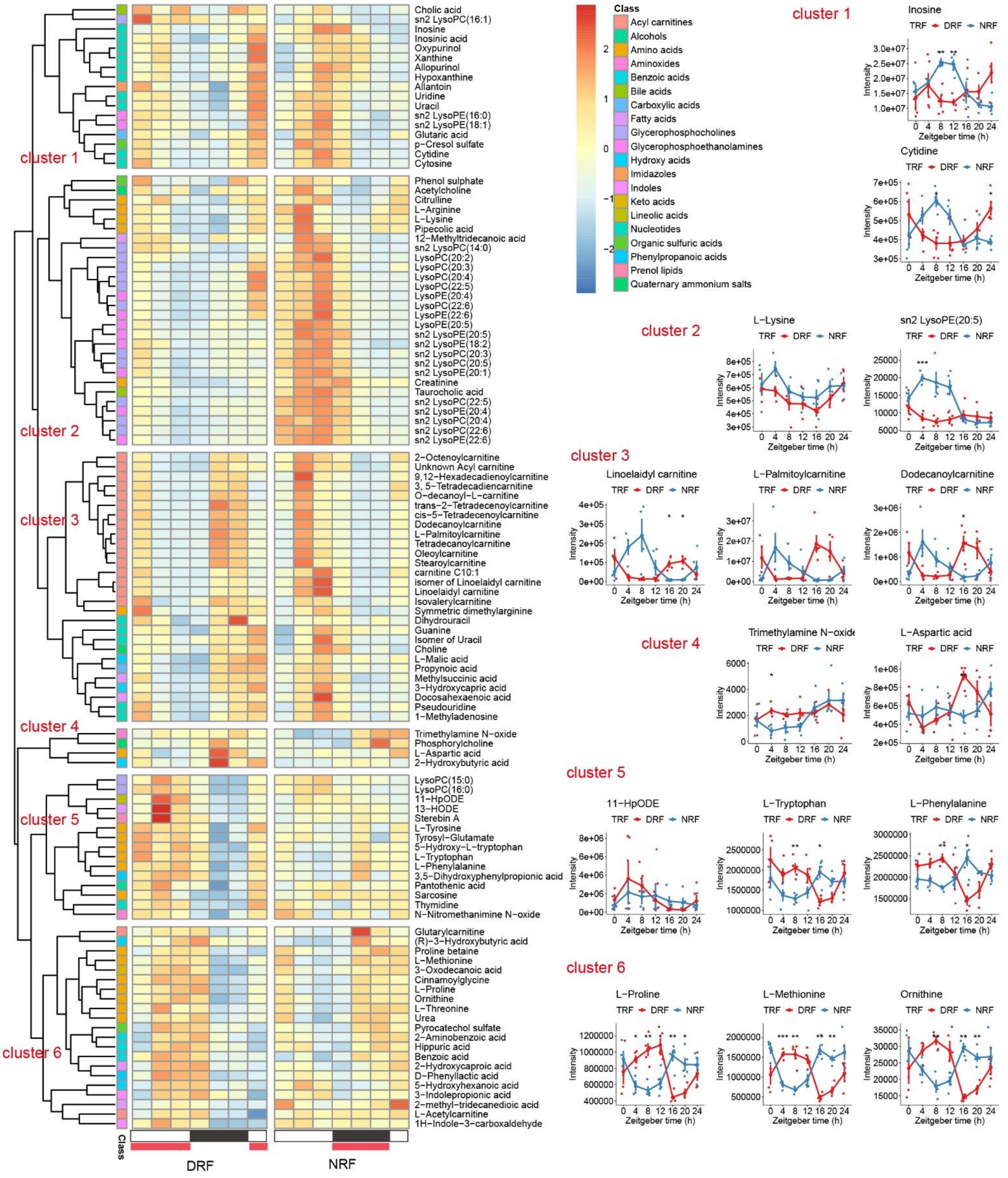

Figure S7. Circadian and Phase Analysis of Cardiac Metabolomes, Related to Figure 7. Heatmap showing diurnal profiles of diurnal metabolites in heart. Euclidean clustering grouped cardiac diurnal metabolites into six clusters. Diurnal profiles of representative

metabolites from the five clusters were shown as mean ± sem (n = 4-5). Multiple t-tests with Bonferroni correction; $P >= 0.05$ is not shown; *$P < 0.05$; **$P < 0.01$, ***$P < 0.001$.

Supplemental Table 6

Oligonucleotides	SOURCE
Primers: Arntl<br>Forward: CTTGCAAGCACCTTCCTTCC<br>Reverse: GGGTCATCTTTGTCTGTGTC	This paper
Primers: Per2<br>Forward: ATGCTCGCCATCCACAAGA<br>Reverse: GCGGAATCGAATGGGAGAAT	This paper
Primers: Nr1d1<br>Forward: TACATTGGCTCTAGTGGCTCC<br>Reverse: CAGTAGGTGATGGTGGGAAGTA	This paper
Primers: Dbp<br>Forward: CGTGGAGGTGCTAATGACCTTT<br>Reverse: CATGGCCTGGAATGCTTGA	This paper
Primers: u36B4<br>Forward: AGATGCAGCAGATCCGCAT<br>Reverse: GTTCTTGCCCATCAGCACC	This paper
Primers: 1110038B12Rik-201<br>Forward: GCACAATGGGATTTGAGGACAC<br>Reverse: GACAAAGGGCTGGCTCTCAT	This paper
Primers: 2410006H16Rik-201<br>Forward: TGCTCTTCTCGCTCGTTGAG<br>Reverse: TTGTCAACGTCCCGTCAGG	This paper
Primers: AC157910.3-202<br>Forward: GTGACTCTGTTCCCTGGTGA<br>Reverse: TGTGGAGTCACTTTGCTGCA	This paper
Primers: Gm45866-201<br>Forward: AAACAGCCACCACACGGTAC<br>Reverse: CCCTTCCAGTTGGCCTTTGA	This paper

Table S6. RT-qPCR Primers Used for Circadian Rhythm Validation, Related to Figure 2, Figure 3, and Figure 4

Transparent Methods

EXPERIMENTAL MODEL AND SUBJECT DETAILS

Animals

Animal experiments were approved by the Laboratory Animal Welfare and Ethics Committee of the Third Military Medical University (TMMU), China. All experiments conform to the relevant regulatory standards of TMMU. Special pathogen-free (SPF) mice were purchased from Hunan SJT Laboratory Animal Co. and housed in a SPF barrier facility. C57BL/6J female or male mice at 6 or 7 weeks of age were grouped housed, and entrained to a 12h light:12h dark cycle with normal chow food and water *ad libitum* during acclimation for at least one week. Animals were randomly assigned in a 1:1 ratio to time-restricted feeding groups. The body weight statistics were balanced between groups before subjected to time-restricted feeding.

METHOD DETAILS

Time-restricted feeding and sample collection

For transcriptome and metabolome profiling

C57BL/6J female mice at 7 weeks of age were grouped housed, and entrained to a 12h light:12h dark cycle (light intensity 200 lux) with normal chow food and water *ad libitum* for at least 7 days before assigned to daytime-restricted feeding (DRF) and nighttime-restricted feeding (NRF) groups for 7 days. The DRF group had access to food for 12 h from ZT0 to ZT12 where ZT0 denotes light on at 9:00 am in China Standard Time (UTC +8). The NRF group had access to food for 12 h from ZT12 to ZT24 (ZT0). At the end of time-restricted feeding, mice were euthanized by cervical dislocation, and subjected to tissue collection every 4 h over 24 h. Visceral adipose tissue, liver, and heart were collected from one batch (n = 4 per group per time-point). Liver and heart were collected from a second batch for metabolomics (n = 5 per group per time-point; liver samples D24_5 and N24_5 could not be identified because of incorrect labels). Kidney were collected from a third batch (n = 4 per group per time-point). Visceral adipose tissue were collected for lipidomics analysis (n = 4 per group per time-point). Tissues were snap frozen in liquid nitrogen and stored in a -80 deg fridge.

Clock entrainment to inverted feeding in male mice

C57BL/6J male mice at 7 weeks of age were grouped housed, and entrained to a 12h light:12h dark cycle (light intensity 500 lux for male, this is within the range of normal indoor illumination) with normal chow food and water *ad libitum* for at least 7 days before assigned to daytime-restricted feeding (DRF) and nighttime-restricted feeding (NRF) groups for 7 days. The DRF group had access to food for 12 h from ZT0 to ZT12 where ZT0 denotes light on at 9:00 am in China Standard Time (UTC +8). The NRF group had access to food for 12 h from ZT12 to ZT24 (ZT0). At the end of time-restricted feeding, mice were euthanized by cervical dislocation, and subjected to tissue collection every 4 h over 24 h. Visceral adipose tissue, liver, kidney and heart were collected (n = 4 per group per time-point). Tissues were snap frozen in liquid nitrogen and stored in a -80 deg fridge.

Long-term regimens of time-restricted feeding

C57BL/6J female mice at 7 weeks of age were grouped housed, and entrained to a 12h

light:12h dark cycle (light intensity 200 lux) with normal chow food and water *ad libitum* for at least 7 days before assigned to daytime-restricted feeding (DRF) and nighttime-restricted feeding (NRF) groups for 36 days. The DRF group had access to food for 12 h from ZT0 to ZT12 where ZT0 denotes light on at 9:00 am in China Standard Time (UTC +8). The NRF group had access to food for 12 h from ZT12 to ZT24 (ZT0). At the end of time-restricted feeding, mice were euthanized by cervical dislocation, and subjected to tissue collection every 4 h over 24 h. Visceral adipose tissue, liver, kidney and heart were collected (n = 4 per group per time-point). Tissues were snap frozen in liquid nitrogen and stored in a -80 deg fridge.

Constant light exposure

Six-week old C57BL/6J female mice were group-housed, acclimated (light intensity 500 lux) for 7 days, exposed to constant light (LL) for 9 days to induce behavioral arrhythmia (Chen et al., 2008), and subjected to DRF and LL for 9 days. At the end of time-restricted feeding, mice were euthanized by cervical dislocation, and subjected to tissue collection every 4 h over 24 h. Visceral adipose tissue, liver, kidney and heart were collected (n = 4 per group per time-point). Tissues were snap frozen in liquid nitrogen and stored in a -80 deg fridge.

Food intake, body weight, and locomotor activity analyses

Mice were housed in groups (n = 4 per cage, n = 7 cages per treatment) and ear-tagged. Body weight was monitored weekly by a digital precision scale (accuracy 0.1 g) for 36 days. Food intake per cage was manually weighed by a digital precision scale (accuracy 0.1 g) on a daily basis for 36 days. Food pellets were layed on the bedding of cages to avoid smashing and weighed 48 h later. Food intake was represented as food consumption per day per mouse (food consumed in grams divided by (2 days x 4 mice per cage)). For locomotor activity analysis, mice were individually housed and acclimated in 12 h light/dark cycle for two weeks in a cage with a running wheel. Wheel running was recorded in 1-min bins and analyzed using ClockLab (Actimetrics, Evanston, IL). Locomotor actograms were double-plotted.

Global Profiling of Transcripts

Strand-specific sequencing of cDNA library from liver

Total RNA was extracted by TRIzol method (Invitrogen). RNA integrity, purity and concentration were assessed by RNA electrophoresis, the NanoPhotometer spectrophotometer, and the Bioanalyzer 2100 system (Thermo Fisher Scientific, MA, USA). All RNA samples passed the quality control. About 100 mg of liver was subjected to homogenization. One sample represents one mouse and four biological replicates were set up for each time point per dietary treatment. In total, there were 56 RNA extractions for preparing hepatic strand-specific cDNA library.

Library construction, RNA sequencing, read mapping, and gene/transcript quantification were performed by Novogene (Beijing, China). A ribosome-depletion workflow was applied to generate the strand-specific cDNA library (Parkhomchuk et al., 2009). Specifically, ribosomal RNAs were removed by Epicenter Ribo-Zero Kit (Epicenter). The remaining RNAs were fragmented (250-300 bp), and reversely transcribed using random hexamers and M-MuLV Reverse Transcriptase (RNase H-). After RNA digestion by RNase H, second strand cDNA synthesis was subsequently performed using dNTP (dTTP replaced by dUTP) and DNA

polymerase I, followed by purification, terminal repair, polyadenylation, adapter ligation, size selection, and degradation of second-strand U-contained cDNA. The clustering of the index-coded samples was performed on a cBot Cluster Generation System using TruSeq PE Cluster Kit v3-cBot-HS (Illumina) according to the manufacturer's instructions. After cluster generation, the library preparations were sequenced on an Illumina Novaseq6000 platform and 150 bp paired-end reads (PE150) were generated. About 10% of the ribosome-depleted transcript profile contains circular RNA (circRNA), which permits to explore diurnal oscillation of circRNAs.

RNA sequencing of cDNA library from VAT and heart

Total RNA was extracted by TRIzol method (Invitrogen). RNA integrity, purity and concentration were assessed by RNA electrophoresis, the NanoPhotometer spectrophotometer, and the Bioanalyzer 2100 system (Thermo Fisher Scientific, MA, USA). All RNA samples passed the quality control. Two lobes of VAT, or the whole heart were subjected to homogenization. One sample represents one mouse and four biological replicates were set up for each time point per dietary treatment. In total, there were 56 RNA extractions for preparing cDNA libraries of visceral adipose or cardiac mRNAs.

Library construction, RNA sequencing, read mapping, and gene/transcript quantification were performed by Novogene (Beijing, China). A total amount of 1 μg RNA per sample was used as input material for the RNA sample preparations. Sequencing libraries were generated using NEBNext® Ultra RNA Library Prep Kit for Illumina (NEB, USA) following manufacturer's recommendations and index codes were added to attribute sequences to each sample. Briefly, mRNA was purified from total RNA using poly-T oligo-attached magnetic beads. Fragmentation was carried out using divalent cations under elevated temperature in NEBNext First Strand Synthesis Reaction Buffer(5X). First strand cDNA was synthesized using random hexamer primer and M-MuLV Reverse Transcriptase (RNase H-). Second strand cDNA synthesis was subsequently performed using DNA Polymerase I and RNase H. Remaining overhangs were converted into blunt ends via exonuclease/polymerase activities. After adenylation of 3' ends of DNA fragments, NEBNext Adaptor with hairpin loop structure were ligated to prepare for hybridization.

In order to select cDNA fragments of preferentially 250~300 bp in length, the library fragments were purified with AMPure XP system (Beckman Coulter, Beverly, USA). Then 3 μl USER Enzyme (NEB, USA) was used with size-selected, adaptor-ligated cDNA at 37°C for 15 min in followed by 5 min at 95 °C before PCR. Then PCR was performed with Phusion High-Fidelity DNA polymerase, Universal PCR primers and Index (X) Primer. At last, PCR products were purified (AMPure XP system) and library quality was assessed on the Agilent Bioanalyzer 2100 system. The clustering of the index-coded samples was performed on a cBot Cluster Generation System using TruSeq PE Cluster Kit v3-cBot-HS (Illumina) according to the manufacturer's instructions. After cluster generation, the library preparations were sequenced on an Illumina Novaseq6000 platform and 150 bp paired-end reads (PE150) were generated.

RNA sequencing of cDNA library from heart (for validation)

Library construction, RNA sequencing, read mapping, and gene/transcript quantification were performed by Biomarker Technologies (Beijing, China). All RNA samples passed the quality control. Whole heart was subjected to homogenization. As a small-scale validation

experiment, heart samples were collected from a different batch of animals of same age, sex, and treatment, except one mouse was assigned per time point. One sample represents one mouse. In total, there were 14 RNA extractions for preparing cDNA libraries of cardiac mRNAs. Total RNAs were extracted by TRIzol method (Invitrogen). RNA concentration/purity and integrity was measured by NanoDrop 2000 (Thermo Fisher Scientific, Wilmington,DE) and the RNA Nano 6000 Assay Kit of the Agilent Bioanalyzer 2100 system (Agilent Technologies, CA, USA). 1 μg RNA per sample was used for library construction. Sequencing libraries were prepared as described in the previous section. The clustering of the index-coded samples was performed on a cBot Cluster Generation System using TruSeq PE Cluster Kit v4-cBot-HS (Illumina) according to the manufacturer's instructions. After cluster generation, the library preparations were sequenced on an Illumina's Novaseq6000 platform and PE150 reads were generated.

RNA sequencing of cDNA library from kidney

Total RNA was extracted by TRIzol method (Invitrogen). RNA integrity, purity and concentration were assessed by RNA electrophoresis, NanoDrop, and the Bioanalyzer 2100 system (Thermo Fisher Scientific, MA, USA). All RNA samples passed the quality control. The left lobe of kidney was subjected to homogenization. One sample represents one mouse and four biological replicates were set up for each time point per dietary treatment. In total, there were 56 RNA extractions for preparing cDNA libraries of renal mRNAs.

Library construction, RNA sequencing, read mapping, and gene/transcript quantification were performed by BGI (Beijing, China). Oligo(dT)-attached magnetic beads were used to purified mRNA. Purified mRNA was fragmented into small pieces with fragment buffer at an appropriate temperature. Then First-strand cDNA was generated using random hexamer-primed reverse transcription, followed by a second-strand cDNA synthesis. Afterwards, A-Tailing Mix and RNA Index Adapters were added by incubating to end repair. The cDNA fragments obtained from previous step were amplified by PCR, and products were purified by Ampure XP Beads, then dissolved in EB solution. The product was validated on the Agilent Technologies 2100 bioanalyzer for quality control. The double stranded PCR products from previous step were heated denatured and circularized by the splint oligo sequence to get the final library. The single strand circle DNA (ssCir DNA) was formatted as the final library. The final library was amplified with phi29 to make DNA nanoball (DNB) which had more than 300 copies of one molecule. DNBs were loaded into the patterned nanoarray and pair end 150 bases reads (PE150) were generated on BGIseq500 platform (BGI-Shenzhen, China).

Untargeted metabolomics

The extraction protocol for sample metabolome were referenced to a previous method (Yuan et al., 2012). Chromatographic separation was performed on a reversed-phase ACQUITY UPLC HSS T3 1.8 μm, 3.0 × 100 mm columns (Waters, Dublin, Ireland) using an ultra-performance LC system (Agilent 1290 Infinity II; Agilent Technologies, Germany). MS was performed using high-resolution mass spectrometry (5600 Triple TOF Plus, AB Sciex, Singapore) equipped with an ESI source. Data were acquired in TOF full scan method with positive and negative ion modes, respectively. Information-dependent acquisition methods were used for MS/MS analyses of metabolome. The collision energy was set at 35 ± 15 eV.

Metabolite identification was compared with standard references, HMDB (http://www.hmdb.ca/) and METLIN (https://metlin.scripps.edu). 45 isotopically-labeled internal standards were spiked into the samples for semi-quantification of metabolites (see Key Resources Table in Mendeley Data).

Metabolite intensities were normalized according to the following rules, and referred to as Intensity (Song et al., 2020). (1) Peak areas of metabolites were counted when internal standards were available; (2) When Step (1) was not feasible due to unavailability of commercial standards, peak areas were corrected with IS of metabolites of the same class, comparable peak intensities, and/or proximity in retention times; (3) Results from Step (2) were evaluated based on relative standard deviation (RSD) values of each metabolite before and after IS correction. Corrected peak areas were adopted if their corresponding RSDs were smaller than that of original areas in quality control samples.

Targeted lipidomics

Lipid extracts from adipose tissue were prepared by a modified Bligh/Dyer method and analyzed (Lu et al., 2019). Samples were resuspended and spiked with appropriate concentrations of isotope-labeled internal standards. All lipidomics analyses were conducted on an Exion UPLC coupled with a SCIEX QTRAP 6500 PLUS system in an ESI mode (curtain gas = 20, ion spray voltage = 5500 V, temperature = 400 degrees, ion source gas 1 = 35, ion source gas 2 = 35). Glycerolipids including diacylglycerols (DAGs) and triacylglycerols (TAGs) were quantified using a modified version of reverse phase LC/MRM. Separation of neutral lipids were achieved on a Phenomenex Kinetex-C18 2.6 µm column (i.d. 4.6x100 mm) using an isocratic mobile phase containing chloroform:methanol:0.1 M ammonium acetate 100:100:4 (v/v/v) at a flow rate of 160 µL/min. Levels of TAGs were estimated by referencing to spiked internal standards of TAG(14:0)3-d5, TAG(16:0)3-d5 and TAG(18:0)3-d5 obtained from CDN isotopes (Quebec, Canada), respectively. DAGs were quantified using d5-DAG(1,3-17:0) and d5-DAG(1,3-18:1) as internal standards from Avanti Polar Lipids. Free cholesterol and cholesteryl esters were analyzed by HPLC-MS/MS in an APCI mode, and estimated by referencing to internal standards, i.e. cholesterol-26,26,26,27,27,27-d6 and cholesteryl-2,2,3,4,4,6-d6 Octadeconate (CDN isotopes). Lipid levels were expressed in nanomole or micromole of lipids per gram of wet tissue.

Acyl-CoA quantification by LC/MS

Extraction of acyl-CoAs from heart tissue was carried out as follows (Woldegiorgis et al., 1985). 300 µL of extraction buffer containing isopropanol, 50 mM KH2PO4, 50 mg/mL BSA (25:25:1 v/v/v) acidified with glacial acetic acid was added to cells. Next, 19:0-CoA was added as an internal standard and lipids were extracted by incubation at 4 °C for 1 h at 1500 rpm. Following this, 300 µL of petroleum ether was added and the sample was centrifuged at 12,000 rpm for 2 min at 4 °C. The upper phase was removed. The samples were extracted two more times with petroleum ether as described above. To the lower phase finally remaining, 5 µL of saturated ammonium sulfate was added followed by 600 µL of chloroform:methanol (1:2 v/v). The sample was then incubated on a thermomixer at 450 rpm for 20 min at 25 °C, followed by centrifugation at 12000 rpm for 5 min at 4 °C. Clean supernatant was transferred to fresh tube and subsequently dried in the SpeedVac under OH mode (Genevac). Dry extracts were

resuspended in appropriate volume of methanol:water (9:1 v/v) prior to liquid chromatography–mass spectrometry (LC–MS) analyses on a Thermofisher U3000 DGLC coupled to Sciex QTRAP 6500 Plus (Lam et al., 2020).

RNA extraction and real-time quantitative PCR

Tissue RNAs were isolated using Eastep® Super Total RNA Extraction Kit (Promega). Complementary DNA (cDNA) was synthesized using the GoScript™ Reverse Transcription Mix (Promega). cDNA was amplified and analyzed using iTaq™ universal SYBR ® Green Supermix (Bio-Rad) and the Bio-Rad CFX96 Real-Time PCR Detection System (Bio-Rad). PCR protocol: Polymerase activation and DNA denaturation at 95 degrees for 3 min; denaturation at 95 degrees for 10 sec; annealing/extension and plate read at 60 degrees for 30 sec; 40 cycles of quantitative PCR. Results were normalized to u36b4. Primer sequences were listed in Key Resource Table. Experiments were repeated at least twice.

QUANTIFICATION AND STATISTICAL ANALYSIS

Bioinformatics for global transcript profiling

Strand-specific sequencing of cDNA library from liver

Raw data (raw reads) of fastq format were firstly processed through in-house perl scripts (Novogene). In this step, clean data (clean reads) were obtained by removing reads containing adapter, reads containing ploy-N and low-quality reads from raw data. At the same time, Q20, Q30 and GC content the clean data were calculated. All the downstream analyses were based on the clean data with high quality. Reference genome and gene model annotation files were downloaded from genome website directly. HISAT2 v2.0.5 (Kim et al., 2015) was used to index the reference genome (mus_musculus_Ensembl_97) and map the paired-end clean reads. using HISAT2 v2.0.5. Transcripts were assembled by StringTie (Pertea et al., 2015), and determined by Cuffmerge, which filters undirectional and short (< 200 nt) transcripts (Trapnell et al., 2012). Noval lncRNA and mRNAs were determined by Cuffcompare (Trapnell et al., 2012). Particularly, novel lncRNA was determined by their lack of annotation in the current database, and lack of protein-coding potential using CPC2, PFAM, CNCI. Transcript/gene read counts were quantified by StringTie (Pertea et al., 2015). The read counts were normalized by TMM (Trimmed Mean of M-values) method by edgeR (v3.30.3) (Robinson et al., 2010), and converted to Wagner's TPM (Transcripts per Million), based on the length of the gene and reads count mapped to a gene (Wagner et al., 2012). Gene/transcripts with TPM > 1 in at least 20% samples were considered as robustly expressed and kept for downstream analysis.

RNA sequencing of cDNA library from VAT and heart

Raw data (raw reads) of fastq format were firstly processed through in-house perl scripts (Novogene). In this step, clean data (clean reads) were obtained by removing reads containing adapter, reads containing ploy-N or low quality reads (> 50% of bases have a Qphred <= 20) from raw data. At the same time, Q20, Q30 and GC content the clean data were calculated. All the downstream analyses were based on the clean data with high quality. Index of the reference genome was built using HISAT2 v2.0.5 and paired-end clean reads were aligned to the reference genome (mus_musculus_Ensembl_97) using HISAT2 v2.0.5 (Kim et al., 2015). Read counts were quantified by featureCounts v1.5.0-p3 (Liao et al., 2014), normalized by TMM

(Trimmed Mean of M-values) method by edgeR (v3.30.3) (Robinson et al., 2010), and converted to Wagner's TPM (Transcripts per Million) (Wagner et al., 2012). Gene with TPM > 1 in at least 20% samples were considered as robustly expressed and kept for downstream analysis.

RNA sequencing of cDNA library from heart (for validation)

Raw data (raw reads) of fastq format were firstly processed through in-house perl scripts (Biomarker Technologies). In this step, clean data (clean reads in the range of 27.2-31.5 million) were obtained by removing reads containing adapter, reads containing ploy-N and low-quality reads from raw data. At the same time, Q20, Q30, GC-content and sequence duplication level of the clean data were calculated.The adaptor sequences and low-quality sequence reads were removed from the data sets. These clean reads were then mapped to the reference genome sequence. Only reads with a perfect match or one mismatch were further analyzed and annotated based on the reference genome (GRCm38/mm10). Hisat2 tools (v2.0.4) were used to map with reference genome. Read counts were quantified by StringTie (Pertea et al., 2015), normalized by TMM (Trimmed Mean of M-values) method by edgeR (v3.30.3) (Robinson et al., 2010), and converted to Wagner's TPM (Transcripts per Million) (Wagner et al., 2012). Gene with TPM > 1 in at least 20% samples were considered as robustly expressed and kept for downstream analysis.

RNA sequencing of cDNA library from kidney

The sequencing data was filtered via SOAPnuke (v1.5.2) by removing reads containing sequencing adapter, reads with a low-quality base ratio (base quality less than or equal to 5) of > 20%, reads with an unknown base ('N' base) ratio of > 5% (Li et al., 2008). Then, clean reads were obtained and stored in FASTQ format. The clean reads were mapped to the reference genome (GRCm38.p6) using HISAT2 (v2.0.4) (Kim et al., 2015). Bowtie2 (v2.2.5) was applied to align the clean reads to the reference coding gene set (Langmead and Salzberg, 2012). Read counts were quantified by RSEM (v1.2.12) (Li and Dewey, 2011), normalized by TMM (Trimmed Mean of M-values) method by edgeR (v3.30.3) (Robinson et al., 2010), and converted to Wagner's TPM (Transcripts per Million) (Wagner et al., 2012). Gene with TPM > 1 in at least 20% samples were considered as robustly expressed and kept for downstream analysis.

Bioinformatics for previous transcript profiling datasets

Data normalization were performed as follows. Microarray intensity files from GSE52333 and GSE87425 were log2 transformed, subtracted by the median of the sample, and universally added to one median (6.514206 and 6.801615, respectively). Microarray intensity data from GSE93903, expressed in log2 values, was subtracted by the median of the sample, and universally added to one median, 5.107950. RNA-seq data from GSE107787, expressed in RPKM, was used for downstream analysis.

Bioinformatics for untargeted metabolomics

Intensity data per group (treatment ~ time point) were checked for Pearson's correlation. The threshold is r >= 0.8 for at least 4 samples per group. In heart samples, N00_2, N04_5,

N08_1, D00_1, and D20_4 were removed. In liver samples, D00_3 and D04_2 were removed. Only one missing value was spotted, i.e. “5-Hydroxyhexanoic acid” in N16_1 in liver, which was replaced by the minimum in that group (N16). Processed data were subjected to circadian rhythmicity analysis as follows.

Bioinformatics for targeted lipidomics

Quantification data per group (treatment ~ time point) were checked for Pearson's correlation. All samples exhibited a r > 0.99 per group, except DRF04_4 in adipose lipidomics study. DRF04_4 was excluded for downstream analysis for its dramatic deviation from the rest of the samples in DRF04. Data were subjected to circadian rhythmicity analysis as below.

Circadian Rhythmicity Analysis

By incorporating results from JTK_Cycle (JTK) and Lomb-Scargle (LS) methods, the MetaCycle::meta2d function was applied to determine circadian rhythmicity of normalized gene/metabolite/circRNA data from global profiling of transcripts, metabolites or lipids (Wu et al., 2016). meta2d p-value was imputed to Fisher's method, and q-value was imputed to Benjamini-Hochberg (BH) multiple testing procedure. Data were not duplicated nor concatenated. The period length under light/dark cycles is set to 24 h. The period length under constant light is set to 20-28h. To capture 12h rhythms, the period length is set to 12 h (transcripts) and 8-28 h (lipids). BH.Q-value < 0.05 is considered as statistical significance for all genes, except lipids, and metabolites (meta2d_p-value < 0.01) and genes from validation studies of heart (meta2d_pvalue < 0.1, the relaxed p-value was meant to acquire more oscillating genes to capture the general pattern).

Rhythmicity analysis for lncRNA transcript dataset was performed as follows. Quantification profile of lncRNAs was subsetted from the transcriptome profile, and subjected to meta2d function. FDR (meta2d_BH.Q) < 0.05 is considered as statistical significance.

Comparison of circadian rhythm parameters (i.e. mesor, amplitude, phase) between two oscillating groups was computed by circaCompare (Parsons et al., 2020). When circaCompare could not be applied, such as period lengths of the two groups are different or at least one group is not oscillating, circadian rhythm parameters were computed by MetaCycle (Wu et al., 2016).

Phase Set Enrichment Analysis

Phase set enrichment analysis (Zhang et al., 2016) was performed on oscillating genes in different tissues to identify phase-clustered pathways from Gene Ontology gene sets (Source files: c5.all.v7.1.symbols.gmt, c5.bp.v7.1.symbols.gmt, c5.cc.v7.1.symbols.gmt, c5.mf.v7.1.symbols.gmt), which are curated in the Molecular Signatures Database (Broad Institute, MA, USA). Mouse gene nomenclature was converted to human gene nomenclature via “Human and Mouse Homology Classes with Sequence information” (curated by http://www.informatics.jax.org). Domain was set from 0 to 24, indicating the minimal and maximal period length is 0 and 24 h, respectively. Minimal and maximal items per set are 10 and 10000, respectively. Q-value < 0.05 is considered as statistical significance.

Cistrome Enrichment Analysis

Chromosomal location of genes was probed for what factors have a significant binding overlap with your peak set and submitted to http://dbtoolkit.cistrome.org/. Species: Mouse mm10. Data type in Cistrome: Transcription factor, chromatin regulator. Peak number of Cistrome sample to use: Top 1k peaks according to peak enrichment. Chromosomal location of genes covered each gene's 10,000 bp region upstream the transcription-start-site and the gene body.

Visualization

Heatmap of expression profile was visualized by R package: pheatmap with row-wise scaling and gene-specific clustering by Euclidean correlation. Sequencing reads of genes and lncRNAs were visualized on Integrative Genomics Viewer (Broad Institute, Boston, MA). Statistical analysis were imputed and visualized via R packages ggpubr or ggplot2, or via Prism 8.0.1 (RT-qPCR).

To determine the logical relations of oscillating gene/metabolite sets between NRF and DRF, VennDiagram analysis was performed (Chen and Boutros, 2011). The intersected group represents gene/metabolites that oscillated in tissues on both NRF and DRF, whose phase was examined. The groups unique to either NRF or DRF represent genes that oscillate exclusively on one restricted feeding regimen. To determine the logical relations of oscillating gene sets identified in different dietary regimens, UpSetR plot was applied to gene sets (R package: UpSetR 1.4.0).

Statistics

Sample size was determined by Guidelines for Genome-Scale Analysis of Biological Rhythms (Hughes et al., 2017). Principal components analysis was conducted with processed omics data by dudi.pca function in R package: ade4, with the number of axes set to 10. Q-value (FDR) < 0.05, p-value < 0.01 (for lipids, and metabolites) or p-value < 0.1 (for validation study of cardiac transcriptome, n = 1 per time point) is considered as reaching statistical significance. Multiple t-tests with Bonferroni correction were performed in R or Prism 8.0.1 (RT-qPCR) as indicated, and $P < 0.05$ is considered as statistical significance.

ADDITIONAL RESOURCES

KEY RESOURCES TABLE

KEY RESOURCES TABLE

REAGENT or RESOURCE	SOURCE	IDENTIFIER
Critical Commercial Assays		
Eastep® Super Total RNA Extraction Kit	Promega	Cat# LS1040
GoScript™ Reverse Transcription Mix	Promega	Cat# A2801
iTaq™ universal SYBR ® Green Supermix	Bio-Rad	Cat# 1725124
Deposited Data		
Raw and analyzed data (RNA-seq)	This paper	GEO: GSE150385
Analyzed data (RNA-seq, metabolomics, lipidomics, code)	This paper	Mendeley Data DOI: 10.17632/mb25x9t4m7.1
Transcriptomics, heart	This paper	CNGBdb：CNP0001640
Transcriptomics, visceral adipose tissue	This paper	CNGBdb：CNP0001638
Transcriptomics, kidney	This paper	CNGBdb：CNP0001605
Transcriptomics, liver	This paper	CNGBdb：CNP0001639
Experimental Models: Cell Lines		
Experimental Models: Organisms/Strains		
Mus musculus, C57BL/6J, male, 6 weeks, special pathogen-free	Hunan SJA Laboratory Animal Co. LTD	http://www.hnsja.com/product/7.html
Mus musculus, C57BL/6J, female, 6-7 weeks, special pathogen-free	Hunan SJA Laboratory Animal Co. LTD	http://www.hnsja.com/product/7.html

Chemicals, Peptides, and Recombinant Proteins		
Chloroform (HPLC grade)	Honeywell	Cat#049-4
Methanol (HPLC grade)	Fisher chemical	Cat#A452-4
Acetonitrile (LCMS grade)	Fisher chemical	Cat#A955-4
Ammonium hydroxide solution	Sigma-Aldrich	Cat#05002-1L
Ammonium acetate	Sigma-Aldrich	Cat#73594
Formic acid (98%)	J&K	Cat#299272
RIPA lysis buffer	Meilunbio	Cat#MA0151
Protease inhibitor cocktail	Sigma-Aldrich	Cat#P8340-5ML
d5-DAG(1,3-17:0)	Avanti Polar Lipids	Cat#110537
d5-DAG(1,3-18:1)	Avanti Polar Lipids	Cat#110581
19:0-CoA	Avanti Polar Lipids	Cat#870738
TAG(14:0)3-d5	CDN Isotopes	Cat#D-6958
TAG(16:0)3-d5	CDN Isotopes	Cat#D-5815
TAG(18:0)3-d5	CDN Isotopes	Cat#D-5816
Cholesterol-26,26,26,27,27,27-d6	CDN Isotopes	Cat#D-2139
Cholesteryl-2,2,3,4,4,6-d6 Octadecanoate	CDN Isotopes	Cat#D-5823
L-Phenylalanine-d8	Cambridge Isotope Laboratories	Cat# DLM-372-1
L-Tryptophan-d8	Cambridge Isotope Laboratories	Cat#DLM-6903-0.25

L-Isoleucine-d10	Cambridge Isotope Laboratories	Cat#DLM-141-0.1
L-Asparagine-13C4	Cambridge Isotope Laboratories	Cat#CLM-8699-H-0.05
L-Methionine-d3	Cambridge Isotope Laboratories	Cat#DLM-431-1
L-Valine-d8	Cambridge Isotope Laboratories	Cat#DLM-311-0.5
L-Proline-d7	Cambridge Isotope Laboratories	Cat#DLM-487-0.1
L-Alanine-d7	Cambridge Isotope Laboratories	Cat#DLM-251-PK
DL-Serine-d3	Cambridge Isotope Laboratories	Cat#DLM-1073-1
L-Phenylalanine-d8	Cambridge Isotope Laboratories	Cat# DLM-372-1
L-Tryptophan-d8	Cambridge Isotope Laboratories	Cat#DLM-6903-0.25
L-Isoleucine-d10	Cambridge Isotope Laboratories	Cat#DLM-141-0.1

L-Asparagine-13C4	Cambridge Isotope Laboratories	Cat#CLM-8699-H-0.05
L-Methionine-d3	Cambridge Isotope Laboratories	Cat#DLM-431-1
L-Valine-d8	Cambridge Isotope Laboratories	Cat#DLM-311-0.5
L-Proline-d7	Cambridge Isotope Laboratories	Cat#DLM-487-0.1
L-Alanine-d7	Cambridge Isotope Laboratories	Cat#DLM-251-PK
DL-Glutamic acid-d5	Cambridge Isotope Laboratories	Cat#DLM-357-0.25
L-Aspartic acid-d3	Cambridge Isotope Laboratories	Cat#DLM-546-0.1
L-Arginine-d7	Cambridge Isotope Laboratories	Cat#DLM-541-0.1
L-Glutamine-d5	Cambridge Isotope Laboratories	Cat#DLM-1826-0.1
L-Lysine-d9	Cambridge Isotope Laboratories	Cat#DLM-570-0.1

L-Histidine-d5	Cambridge Isotope Laboratories	Cat#DLM-7855
Taurine-13C 2	Cambridge Isotope Laboratories	Cat#CLM-6622-0.25
Betaine-d11	Cambridge Isotope Laboratories	Cat#DLM-407-1
Urea-(13C,15N2)	Cambridge Isotope Laboratories	Cat#CLM-234-0.5
L-lactate-13C3	Sigma-Aldrich	Cat#485926-500MG
Trimethylamine N-oxide-d9	Cambridge Isotope Laboratories	Cat#DLM-4779-1
Choline-d10	Cambridge Isotope Laboratories	Cat#DLM-141-0.1
Malic acid-d3	Cambridge Isotope Laboratories	Cat#DLM-9045-0.1
Citric acid-d4	Cambridge Isotope Laboratories	Cat#DLM-3487-0.5
Succinic acid-d4	Cambridge Isotope Laboratories	Cat#DLM-584-1
Fumaric acid-d4	Cambridge Isotope Laboratories	Cat#DLM-7654-1

Hypoxanthine-d3	Cambridge Isotope Laboratories	Cat#DLM-2923-0.1
Xanthine-15N2	Cambridge Isotope Laboratories	Cat#NLM-1698-0.1
Thymidine (13C10,15N2)	Cambridge Isotope Laboratories	Cat#CNLM-3902-25
Inosine-15N4	Cambridge Isotope Laboratories	Cat#NLM-4264-0.01
Cytidine-13C5	Cambridge Isotope Laboratories	Cat#CLM-3679-0.05
Uridine-d2	Cambridge Isotope Laboratories	Cat#DLM-7693-0.05
Methylsuccinic acid-d6	Cambridge Isotope Laboratories	Cat#DLM-2960-1
Benzoic acid-d5	Cambridge Isotope Laboratories	Cat#DLM-122-1
Creatine-d3	Cambridge Isotope Laboratories	Cat#DLM-1302-0.25
Creatinine-d3	Cambridge Isotope Laboratories	Cat#DLM-3653-0.1

Glutaric acid-d4	Cambridge Isotope Laboratories	Cat#DLM-3106-5
Glycine-d	Cambridge Isotope Laboratories	Cat# DLM-1674-5
Kynurenic acid-d5	Cambridge Isotope Laboratories	Cat#DLM-7374-PK
L-Citrulline-d4	Cambridge Isotope Laboratories	Cat#DLM-6039-0.01
L-Threonine-(13C4,15N)	Cambridge Isotope Laboratories	Cat#CNLM-587-0.1
L-Tyrosine-d7	Cambridge Isotope Laboratories	Cat#DLM-589-0.05
P-cresol sulfate-d7	Cambridge Isotope Laboratories	Cat#DLM-9786-0.01
Sarcosine-d3	Cambridge Isotope Laboratories	Cat#DLM-6874-0.1
Trans-4-hydroxy-L-proline-d3	Cambridge Isotope Laboratories	Cat#DLM-9778-PK
Uric acid-(13C; 15N3)	Cambridge Isotope Laboratories	Cat#CNLM-10617-0.001
Software and Algorithms		

R Project for Statistical Computing	https://www.r-project.org	V4.0.2<br>RRID:SCR_001905
RStudio	http://www.rstudio.com	v1.2.5033
R package: edgeR	(Robinson et al. 2010)	v3.30.3
R package: MetaCycle	(Wu et al., 2016)	v1.2.0<br>https://github.com/gangwug/MetaCycle
R package: circacompare	(Parsons et al., 2020)	v0.1.0
R package: ade4	(Dray and Dufour, 2007)	v1.7-16
R package: ggpubr	https://www.rdocumentation.org/packages/ggpubr	v0.2.5
R package: ggplot2	https://cran.r-project.org/web/packages/ggplot2/index.html	RRID:SCR_014601
R package: dplyr	https://cran.r-project.org/web/packages/dplyr/index.html	RRID:SCR_016708
R package: VennDiagram	(Chen and Boutros, 2011)	v1.6.20<br>https://cran.r-project.org/web/packages/VennDiagram/index.html

R package: pheatmap	https://cran.r-project.org/web/packages/pheatmap/index.html	v1.0.12
R package: UpSetR	https://cran.r-project.org/web/packages/UpSetR/	v1.4.0
HISAT2	(Kim et al., 2015)	http://ccb.jhu.edu/software/hisat2
StringTie	(Pertea et al., 2015)	https://ccb.jhu.edu/software/stringtie/
featureCount	(Liao et al., 2014)	1.5.0-p3<br>http://subread.sourceforge.net/
Cufflinks	(Trapnell et al., 2012)	http://cole-trapnell-lab.github.io/cufflinks/
find_circ	(Memczak et al., 2013)	https://github.com/marvin-jens/find_circ
rMATS	(Shen et al., 2014)	http://rnaseq-mats.sourceforge.net/index.html
CIRI	(Gao et al., 2015)	https://sourceforge.net/projects/ciri/
Phase Set Enrichment Analysis	(Zhang et al., 2016)	https://github.com/ranafi/PSEA
Integrative Genomics Viewer (IGV)	http://software.broadinstitute.org/software/igv/home	v2.8.0

Mendeley Desktop	https://www.mendeley.com/?interaction_required=true	v1.19.4
Excel 2019	Microsoft	N/A
Graphpad Prism 8.0.1	https://www.graphpad.com	v8.0.1
Other		
Normal chow diet (Rodent maintenance diet)	Hunan SJA Laboratory Animal Co. LTD	http://www.hnsja.com/product/13.html

Supplemental References

Chen, H., Boutros, P.C., 2011. VennDiagram: a package for the generation of highly-customizable Venn and Euler diagrams in R. BMC Bioinformatics 12, 35. https://doi.org/10.1186/1471-2105-12-35

Chen, R., Seo, D.O., Bell, E., Von Gall, C., Lee, D.C., 2008. Strong resetting of the mammalian clock by constant light followed by constant darkness. J. Neurosci. 28, 11839–11847. https://doi.org/10.1523/JNEUROSCI.2191-08.2008

Dray, S., Dufour, A.-B., 2007. The ade4 Package: Implementing the Duality Diagram for Ecologists. J. Stat. Softw. 22. https://doi.org/10.18637/jss.v022.i04

Gao, Y., Wang, J., Zhao, F., 2015. CIRI: An efficient and unbiased algorithm for de novo circular RNA identification. Genome Biol. 16, 1–16. https://doi.org/10.1186/s13059-014-0571-3

Kim, D., Langmead, B., Salzberg, S.L., 2015. HISAT: a fast spliced aligner with low memory requirements. Nat. Methods 12, 357–360. https://doi.org/10.1038/nmeth.3317

Lam, S.M., Zhou, T., Li, J., Zhang, S., Chua, G.H., Li, B., Shui, G., 2020. A robust, integrated platform for comprehensive analyses of acyl-coenzyme As and acyl-carnitines revealed chain length-dependent disparity in fatty acyl metabolic fates across Drosophila development. Sci. Bull. 65, 1840–1848. https://doi.org/10.1016/j.scib.2020.07.023

Langmead, B., Salzberg, S.L., 2012. Fast gapped-read alignment with Bowtie 2. Nat. Methods 9, 357–359. https://doi.org/10.1038/nmeth.1923

Li, B., Dewey, C.N., 2011. RSEM: accurate transcript quantification from RNA-Seq data with or without a reference genome. BMC Bioinformatics 12, 323. https://doi.org/10.1186/1471-2105-12-323

Li, R., Li, Y., Kristiansen, K., Wang, J., 2008. SOAP: short oligonucleotide alignment program. Bioinformatics 24, 713–714. https://doi.org/10.1093/bioinformatics/btn025

Liao, Y., Smyth, G.K., Shi, W., 2014. featureCounts: an efficient general purpose program for

assigning sequence reads to genomic features. Bioinformatics 30, 923–930. https://doi.org/10.1093/bioinformatics/btt656

Lu, J., Lam, S.M., Wan, Q., Shi, L., Huo, Y., Chen, L., Tang, X., Li, B., Wu, X., Peng, K., Li, M., Wang, S., Xu, Y., Xu, M., Bi, Y., Ning, G., Shui, G., Wang, W., 2019. High-Coverage Targeted Lipidomics Reveals Novel Serum Lipid Predictors and Lipid Pathway Dysregulation Antecedent to Type 2 Diabetes Onset in Normoglycemic Chinese Adults. Diabetes Care 42, 2117–2126. https://doi.org/10.2337/dc19-0100

Memczak, S., Jens, M., Elefsinioti, A., Torti, F., Krueger, J., Rybak, A., Maier, L., Mackowiak, S.D., Gregersen, L.H., Munschauer, M., Loewer, A., Ziebold, U., Landthaler, M., Kocks, C., Le Noble, F., Rajewsky, N., 2013. Circular RNAs are a large class of animal RNAs with regulatory potency. Nature 495, 333–338. https://doi.org/10.1038/nature11928

Parkhomchuk, D., Borodina, T., Amstislavskiy, V., Banaru, M., Hallen, L., Krobitsch, S., Lehrach, H., Soldatov, A., 2009. Transcriptome analysis by strand-specific sequencing of complementary DNA. Nucleic Acids Res. 37, e123–e123. https://doi.org/10.1093/nar/gkp596

Parsons, Rex, Parsons, Richard, Garner, N., Oster, H., Rawashdeh, O., 2020. CircaCompare: A method to estimate and statistically support differences in mesor, amplitude and phase, between circadian rhythms. Bioinformatics 36, 1208–1212. https://doi.org/10.1093/bioinformatics/btz730

Pertea, M., Pertea, G.M., Antonescu, C.M., Chang, T.C., Mendell, J.T., Salzberg, S.L., 2015. StringTie enables improved reconstruction of a transcriptome from RNA-seq reads. Nat. Biotechnol. 33, 290–295. https://doi.org/10.1038/nbt.3122

Robinson, M.D., McCarthy, D.J., Smyth, G.K., 2010. edgeR: a Bioconductor package for differential expression analysis of digital gene expression data. Bioinformatics 26, 139–140. https://doi.org/10.1093/bioinformatics/btp616

Shen, S., Park, J.W., Lu, Z.X., Lin, L., Henry, M.D., Wu, Y.N., Zhou, Q., Xing, Y., 2014. rMATS: Robust and flexible detection of differential alternative splicing from replicate RNA-Seq data. Proc. Natl. Acad. Sci. U. S. A. https://doi.org/10.1073/pnas.1419161111

Song, J.W., Lam, S.M., Fan, X., Cao, W.J., Wang, S.Y., Tian, H., Chua, G.H., Zhang, C., Meng, F.P., Xu, Z., Fu, J.L., Huang, L., Xia, P., Yang, T., Zhang, S., Li, B., Jiang, T.J., Wang, R., Wang, Z., Shi, M., Zhang, J.Y., Wang, F.S., Shui, G., 2020. Omics-Driven Systems Interrogation of Metabolic Dysregulation in COVID-19 Pathogenesis. Cell Metab. 32, 188-202.e5. https://doi.org/10.1016/j.cmet.2020.06.016

Trapnell, C., Roberts, A., Goff, L., Pertea, G., Kim, D., Kelley, D.R., Pimentel, H., Salzberg, S.L., Rinn, J.L., Pachter, L., 2012. Differential gene and transcript expression analysis of RNA-seq experiments with TopHat and Cufflinks. Nat. Protoc. 7, 562–578. https://doi.org/10.1038/nprot.2012.016

Wagner, G.P., Kin, K., Lynch, V.J., 2012. Measurement of mRNA abundance using RNA-seq data: RPKM measure is inconsistent among samples. Theory Biosci. 131, 281–285. https://doi.org/10.1007/s12064-012-0162-3

Woldegiorgis, G., Spennetta, T., Corkey, B.E., Williamson, J.R., Shrago, E., 1985. Extraction of tissue long-chain acyl-CoA esters and measurement by reverse-phase high-performance liquid chromatography. Anal. Biochem. 150, 8–12. https://doi.org/10.1016/0003-2697(85)90434-8

Wu, G., Anafi, R.C., Hughes, M.E., Kornacker, K., Hogenesch, J.B., 2016. MetaCycle: An integrated R package to evaluate periodicity in large scale data. Bioinformatics 32, 3351–3353. https://doi.org/10.1093/bioinformatics/btw405

Yuan, M., Breitkopf, S.B., Yang, X., Asara, J.M., 2012. A positive/negative ion-switching, targeted mass spectrometry-based metabolomics platform for bodily fluids, cells, and fresh and fixed tissue. Nat. Protoc. 7, 872–881. https://doi.org/10.1038/nprot.2012.024

Zhang, R., Podtelezhnikov, A.A., Hogenesch, J.B., Anafi, R.C., 2016. Discovering Biology in Periodic Data through Phase Set Enrichment Analysis (PSEA). J. Biol. Rhythms 31, 244–257. https://doi.org/10.1177/0748730416631895
